# Memory effects of climate and vegetation affecting net ecosystem CO_2_ fluxes in global forests

**DOI:** 10.1371/journal.pone.0211510

**Published:** 2019-02-06

**Authors:** Simon Besnard, Nuno Carvalhais, M. Altaf Arain, Andrew Black, Benjamin Brede, Nina Buchmann, Jiquan Chen, Jan G. P. W Clevers, Loïc P. Dutrieux, Fabian Gans, Martin Herold, Martin Jung, Yoshiko Kosugi, Alexander Knohl, Beverly E. Law, Eugénie Paul-Limoges, Annalea Lohila, Lutz Merbold, Olivier Roupsard, Riccardo Valentini, Sebastian Wolf, Xudong Zhang, Markus Reichstein

**Affiliations:** 1 Department for Biogeochemical Integration, Max-Planck-Institute for Biogeochemistry, Jena, Germany; 2 Laboratory of Geo-Information Science and Remote Sensing, Wageningen University, Netherlands; 3 CENSE, Departamento de Ciências e Engenharia do Ambiente, Faculdade de Ciências e Tecnologia, Universidade NOVA de Lisboa, Caparica, Portugal; 4 School of Geography and Earth Sciences and McMaster Center For Climate Change, McMaster University, Hamilton, Ontario, Canada; 5 Faculty of Land and Food Systems, University of British Columbia, Vancouver, Canada; 6 ETH Zurich, Department of Environmental Systems Sciences, Zurich, Switzerland; 7 CGCEO/Geography, Michigan State University, East Lansing, MI, United States of America; 8 National Commission for the Knowledge and Use of Biodiversity (CONABIO), Mexico City, México; 9 Laboratory of Forest Hydrology, Graduate School of Agriculture, Kyoto University, Kyoto, Japan; 10 Faculty of Forest Sciences, University of Goettingen, Göttingen, Germany; 11 College of Forestry, Oregon State University, Corvallis, OR, United States of America; 12 Finnish Meteorological Institute, Helsinki, Finland; 13 Mazingira Centre, International Livestock Research Institute (ILRI), Nairobi, Kenya; 14 CIRAD, UMR Eco&Sols, LMI IESOL, Dakar, Senegal; 15 Eco&Sols, University Montpellier, CIRAD, INRA, IRD, Montpellier SupAgro, Montpellier, France; 16 University of Tuscia, Department for Innovation on Biological, Agro-food and Forest Systems (DIBAF), Viterbo, Italy; 17 ETH Zurich, Department of Environmental Systems Science, Physics of Environmental Systems, Zurich, Switzerland; 18 Research Institute of Forestry, Chinese Academy of Forestry, Haidian District, Beijing, P.R.China; Tennessee State University, UNITED STATES

## Abstract

Forests play a crucial role in the global carbon (C) cycle by storing and sequestering a substantial amount of C in the terrestrial biosphere. Due to temporal dynamics in climate and vegetation activity, there are significant regional variations in carbon dioxide (CO_2_) fluxes between the biosphere and atmosphere in forests that are affecting the global C cycle. Current forest CO_2_ flux dynamics are controlled by instantaneous climate, soil, and vegetation conditions, which carry legacy effects from disturbances and extreme climate events. Our level of understanding from the legacies of these processes on net CO_2_ fluxes is still limited due to their complexities and their long-term effects. Here, we combined remote sensing, climate, and eddy-covariance flux data to study net ecosystem CO_2_ exchange (NEE) at 185 forest sites globally. Instead of commonly used non-dynamic statistical methods, we employed a type of recurrent neural network (RNN), called Long Short-Term Memory network (LSTM) that captures information from the vegetation and climate’s temporal dynamics. The resulting data-driven model integrates interannual and seasonal variations of climate and vegetation by using Landsat and climate data at each site. The presented LSTM algorithm was able to effectively describe the overall seasonal variability (Nash-Sutcliffe efficiency, NSE = 0.66) and across-site (NSE = 0.42) variations in NEE, while it had less success in predicting specific seasonal and interannual anomalies (NSE = 0.07). This analysis demonstrated that an LSTM approach with embedded climate and vegetation memory effects outperformed a non-dynamic statistical model (i.e. Random Forest) for estimating NEE. Additionally, it is shown that the vegetation mean seasonal cycle embeds most of the information content to realistically explain the spatial and seasonal variations in NEE. These findings show the relevance of capturing memory effects from both climate and vegetation in quantifying spatio-temporal variations in forest NEE.

## Introduction

Forests cover about 30% of the terrestrial surface of our planet, accounting for 75% of gross primary production (GPP), and store 45% of all terrestrial carbon (C) [1–3]. This fundamental role highlights the importance of understanding forest C dynamics, which are generally driven by climatic conditions and vegetation dynamics as well as natural and anthropogenic disturbances [4–6]. Changes in climate and disturbance regime can influence the development, structure, and functioning of forest ecosystems [7–12], therefore causing anomalies in the net carbon dioxide (CO_2_) exchange of terrestrial ecosystems (NEE). As a result, quantifying the effects of climatic variations and forest disturbances on biosphere-atmosphere CO_2_ fluxes across-scales has considerable importance for understanding the net CO_2_ balance of forest ecosystems [13–16].

Disturbances, such as fire, disease, insect outbreaks, drought, windthrow, or harvesting, can shift forest ecosystems into early stages of ecological succession [17, 18]. These events can potentially trigger an accelerated release of stored C back to the atmosphere by reducing the amount of photosynthetic tissue and also by increasing the pool of respiring detritus material for subsequent gradual release [14, 19–21]. During recovery, forests accumulate biomass and potentially sequester C from the atmosphere at rates that could alter current trends of atmospheric C cycling [10]. Post-disturbance successional trajectories are often complex, depending on pre-disturbance forest structure and function, disturbance type, frequency, and intensity [22, 23] as well as the climate of the region [24, 25] and land management. Some disturbances, such as insect outbreaks, can slow down recovery process during regeneration or transform forests from closed to open canopies [26], while other low to moderate severity disturbances increase structural complexity leading to less of an impact on mid-succession net primary productivity than is often assumed [27]. Therefore, climate and disturbance regimes contribute to interannual and seasonal variations in forest net CO_2_ fluxes. Changes in climate may also exacerbate the frequency and intensity of extreme meteorological events (e.g. droughts, [6] or associated fire events [28, 29]), thereby increasing both mortality rates and the vulnerability of forest ecosystems [30], which would necessarily impact the dynamics of ecosystem C cycle.

Current response patterns observed in forest CO_2_ fluxes depend on the contemporaneous environment conditions as well as on the so-called memory effects of disturbances, climatic variation, and their interactions [30, 31]. In fact, disturbances or climate extreme events exert both instantaneous and lagged impacts on biosphere-atmosphere C fluxes [6, 32]. Memory effects can be defined as the influence that past events have on the present or future responses of an ecosystem to environmental conditions. Extensive research has been done to understand climate and disturbance memory effects on CO_2_ fluxes (i.e. NEE, gross primary productivity, and ecosystem respiration) [33–38]. However, given the complexity of NEE responses to disturbances and climate extremes and highly non-linear processes, the legacies of these events on CO_2_ fluxes remain unclear [32, 39], and thus they are rarely implemented in current C cycle models. As such, statistical models capable of dynamically incorporating temporal information related to episodic disturbances and climatic fluctuations are required to enhance our understanding and predictive capabilities of the global C budget [8, 40]. Recently, deep learning (DL) techniques, such as Recurrent Neural Networks (RNNs), have shown the potential to capture long-term temporal dependencies and variable-length observations [41–43]. Yet, DL is early in its application for CO_2_ flux predictions [44]; questions related to the potential of extracting temporal information for estimating CO_2_ fluxes across-scales have yet to be investigated.

In this study, we explore the potential of a dynamic statistical approach—a type of RNN, called Long Short-Term Memory model (LSTM)—to characterize the memory effects of disturbance and climate variations on NEE across temporal and spatial scales at 185 forest and woodland FLUXNET sites globally utilizing remote sensing, climate, and eddy-covariance (EC) flux datasets. In particular, this study focuses on: (1) comparing the statistical power of an LSTM approach to a Random Forest algorithm in predicting ecosystem level NEE, and (2) assessing the importance of capturing the memory effects of vegetation and climate to predict forest NEE using data-driven LSTMs. The analysis focuses on the variations in NEE spatially and temporally for seasonal, monthly, and interannual anomalies, for which a factorial experiment was designed as explained below. We propose that the application of dynamic statistical approaches results in estimating net CO_2_ fluxes across-scales more realistically by including the responses of NEE to antecedent climate and disturbance conditions.

## Materials and methods

### Data materials

#### Eddy-covariance data and quality check

The current dataset consists of 185 forest and woodland sites (S1 Table) composed of five plant functional types (PFTs): deciduous broadleaf forest (DBF; n = 42), deciduous needleleaf forest (DNF; n = 1), evergreen broadleaf forest (EBF; n = 27), evergreen needleleaf forest (ENF; n = 81), mixed forest (MF; n = 14), woody savanna (WSA; n = 10), and savanna (SAV, n = 10); and four climate class: arid (n = 11), boreal (n = 67), temperate (n = 86), and tropical (n = 21). We aggregated DBF and DNF into a deciduous forest class, EBF and ENF into an evergreen forest class, and SAV and WSA into a savanna class. The sites were part of both version 2 of the LaThuile FLUXNET and the FLUXNET2015 datasets (https://fluxnet.fluxdata.org) of the FLUXNET network [45, 46]. For each site, continuously measured or gap-filled net CO_2_ fluxes (i.e. NEE) and microclimatic variables (i.e. air temperature (T_*air*_), precipitation (P), global radiation (Rg), and vapor pressure deficit (VPD)) were obtained at half-hourly time intervals from the FLUXNET datasets. The data processing included: storage-correction despiking, u_*_-filtering [47], flux partitioning [48]. Half-hourly NEE were aggregated into monthly averages (i.e. seasonal cycle). Only monthly observations with more than 80% of the original or good quality gap-filled data were considered in this analysis [47]. A total of ≃ 14, 000 observed or gapfilled monthly NEE flux data was used, from which ≃ 1, 500 monthly observations were collected in forests younger than 30 years.

#### Remote sensing data

**Fig 1:**
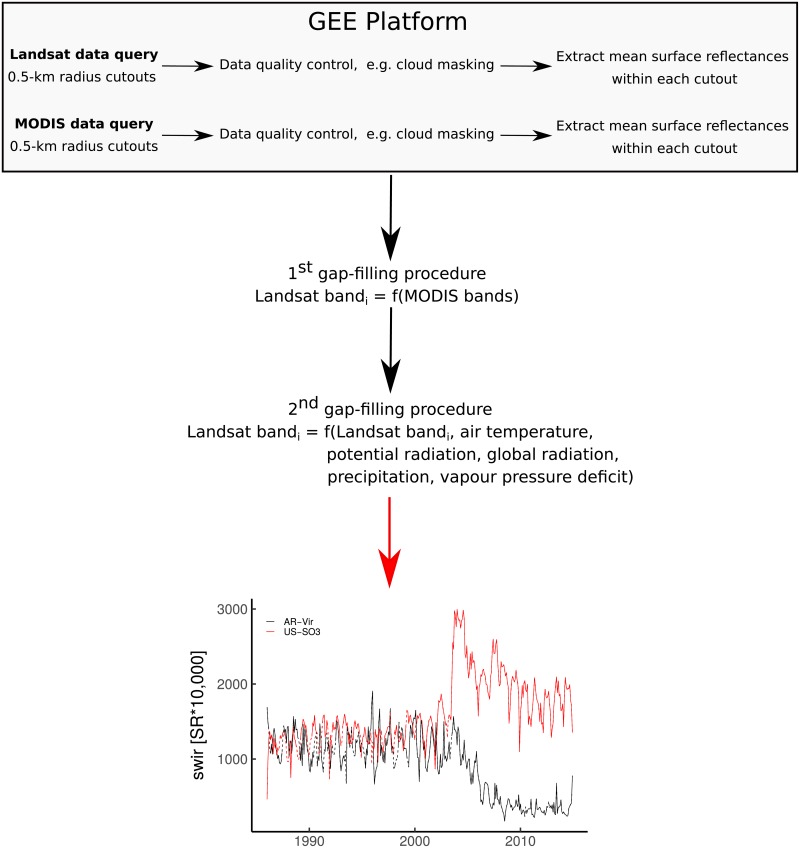
Flowchart of the Landsat data extraction and post-processing. SWIR = Shortwave Infrared. SR = Surface Reflectance. Monthly temporal gap-filled Landsat time series from 1982 to 2015 of the shortwave Infrared band are shown for AR-Vir and US-SO3 sites where, respectively, afforestation-reforestation and fire followed by a regrowth were reported in 2003. The solid and the dashed lines depict the real observations and the gap-filled data, respectively.

For each FLUXNET site, the entire multi-temporal collection 1 from the Landsat 4, 5, 7 and 8 archives (https://www.usgs.gov/) spanning the past 30 years at 30 meters resolution was collected. Landsat data have been pre-processed using the Landsat Ecosystem Disturbance Adaptive Processing System (LEDAPS) [49] and the Landsat Surface Reflectance Code (LaSRC) (https://landsat.usgs.gov/landsat-surface-reflectance-data-products) for atmospheric correction. Poor quality retrievals due to the clouds, cloud shadows, snow, and ice were masked out [50, 51]. The data extraction and the pre-processing chains (i.e. cloud, cloud shadow masking, and downloading) were implemented in the Google Earth Engine (GEE) platform [52] (https://earthengine.google.com/). The seven spectral bands of the Landsat product were used; i.e. blue, green, red, near infrared (NIR), shortwave infrared (SWIR) 1, SWIR 2, and thermal infrared (TIR) (https://landsat.usgs.gov/what-are-band-designations-landsat-satellites). To better represent the EC footprint area, a circular buffer of 500 m radius centered on each FLUXNET tower was defined for which a mean value within the different Landsat cutouts was extracted. Note that the proposed LSTM approach can only be implemented with regular time series, but most of the Landsat time series were irregular due to cloud cover or data quality issues. A first gap-filling procedure was conducted by predicting monthly reflectance values for each Landsat band with a Random Forest (RF) model [53, 54] using the monthly Moderate Resolution Imaging Spectroradiometer (MODIS, MCD43A4 version 6) bands as predictive variables (S1 Fig). The gap-filling procedure was completed for the remaining gaps in the entire Landsat time series (i.e. from the 1980s to now) by predicting each Landsat band with an RF model using climate variable (i.e. T_*air*_, Precip, Rg, VPD, rpot), PFT, month of the year, and latitude as predictive variables (Fig 1 and S2 Fig). For the two aforementioned gap-filling procedures, the best set of the predictors for predicting each Landsat band independently was obtained with a feature selection analysis (i.e. the Boruta algorithm [55]).

#### Climate data

Long-term time series of T_*air*_, P, Rg, and VPD were down-scaled for the period of 1979-2015 from the ERA-Interim datasets [56]. For each site, the three nearest grid cells in the ERA-Interim datasets were extracted and several statistical models were trained (i.e. relational logistic regression, kernel ridge regression, Gaussian processes regression, and neural networks) for each target variable (i.e. T_*air*_, P, Rg, and VPD) using the time series of the three nearest gridcells as predictive variables. For each target variable and at each site, the best statistical model was consequently selected based on the highest Nash-Sutcliffe modeling efficiency (NSE) (S3 Fig). These down-scaled climate time series were used to gap-fill climate observations measured at the site level in order to have climatic data covering the entire remote sensing data period.

### Recurrent neural network model for dynamic modeling

RNNs were employed to learn vegetation and climate history based on sequential observations(https://github.com/bgi-jena/RNNFluxes.jl.git) [44]. RNNs are effective tools for capturing temporal information from sequential/time series data. We used a special kind of RNNs, capable of learning long-term dependencies, called long short-term memory networks (LSTMs) [57]. LSTMs utilize relevant information from all previous observations and are suitable to model long-term temporal dependencies and memory effects.

**Fig 2:**
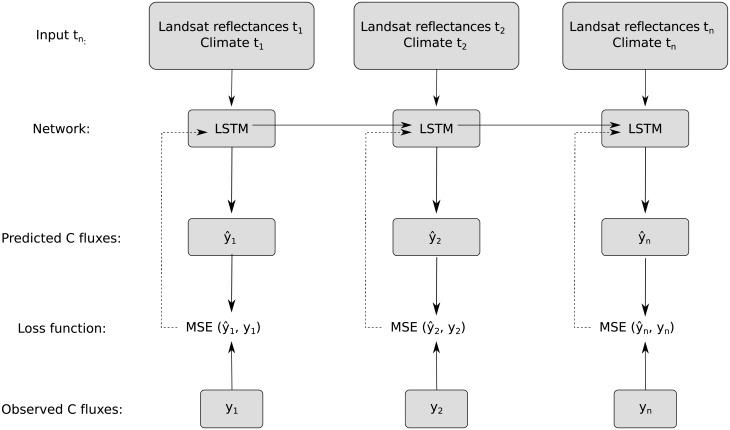
Flowchart of the proposed LSTM approach. Figure adapted from [61]. Each individual timestep is a monthly observation for the period 1982 to 2015. Landsat surface reflectances correspond to the seven spectral bands of the Landsat product; i.e. blue, green, red, near infrared, shortwave infrared 1, shortwave infrared 2, and thermal infrared. Climate corresponds to air temperature, precipitation, global radiation, and vapor pressure deficit. At each time step, an LSTM layer containing a set of cells or hidden neurons (10, 20, or 30) processes information based on the input of the current and of all previous observations. Predictions of net ecosystem exchange were performed at each monthly timestep by using information from both current and previous observations. The loss function was only calculated when net ecosystem exchange observations were available; i.e. measurement periods of LaThuile and FLUXNET2015 datasets.

Monthly climate data (i.e. T_*air*_, P, Rg, and VPD) and Landsat raw bands from the period of 1982 to 2015 were used to train the LSTM models (Fig 2). A single layered LSTM was used to learn information based on the input of the current and of all previous observations. At each training iteration, a loss function (Mean Squared Error) was calculated by comparing monthly predicted and observed NEE. The loss function was used to derive the gradients for backpropagation over the entire sequence [58]. The gradients were further used by an *Adam* optimizer [59] for adjusting the weights of the connections in the model so as to minimize the loss function. During the learning procedure, 20% of the training set (i.e. evaluation set) served for optimizing the weights of the networks. The learning procedure was stopped when the calculated loss function on the evaluation set did not decrease after 500 iterations (i.e. early stopping). Additionally, there was a grid search of the LSTM’s hyperparameters; i.e. learning rate (0.1 or 0.01), number of hidden neurons (10, 20, or 30), and dropout (0 or 0.5) [60] to select the optimal set of hyperparameters. Due to the random initialization of LSTMs, 50 runs for each model set-up were performed to assess the uncertainty of the model outputs.

### Experimental design

#### Model set-ups

**Fig 3:**
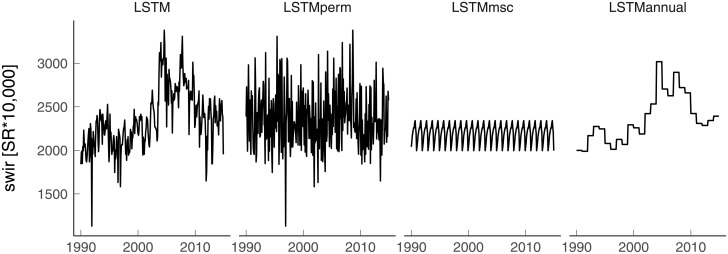
Illustration of the different Landsat time series temporal architectures of the different LSTM model set-ups for the SWIR band only for the period 1990-2015. SWIR = Short Wave Infrared. SR = Surface Reflectance. The US-SO3 site where fire followed by regrowth was reported in 2003 is shown.

In order to understand vegetation and climate memory effects on NEE, a trained LSTM with monthly climatic data (i.e. T_*air*_, P, Rg, and VPD) and monthly Landsat data (i.e. blue, green, red, NIR, SWIR 1, SWIR 2, and TIR bands) was benchmarked against a series of different model set-ups (Table 1 and Fig 3). A comparison of the following five distinct experimental set-ups was performed: (1) LSTM model using the full depth of the Landsat time series and climate data (hereafter *LSTM*); (2) *LSTM* model where the orders of the predictor-target pairs were randomly permuted so that the instantaneous link between dependent and independent variables were kept but the realistic temporal sequences were destroyed (hereafter *LSTM_perm_*); (3) *LSTM* model where the Landsat time series for each band were replaced by their mean seasonal cycle (i.e. mean of each month over the Landsat time series period) while using the actual values of T_*air*_, P, Rg, and VPD (hereafter *LSTM_msc_*); (4) *LSTM* model where the Landsat time series for each band were replaced by their annual mean (i.e. mean of the monthly observations within each year over the Landsat time series period) while using the actual values of T_*air*_, P, Rg, and VPD (hereafter *LSTM_annual_*), and (5) a Random Forest (RF) model [53, 54] using the actual values of the Landsat time series and climate data (hereafter *RF*).

**Table 1:** Design of the factorial experiment. X means that the variant was used to study the respective topic of each row. *LSTM* = LSTM model using the full depth of the Landsat time series and climate data; *LSTM_perm_* = *LSTM* model but the temporal patterns of both the predictive and the target variables were randomly permuted while instantaneous relationships between predictive and target variables were kept; *LSTM_msc_* = *LSTM* model but the Landsat time series for each band were replaced by their mean seasonal cycle, while using the actual values of air temperature (T_*air*_), precipitation (P), global radiation (Rg), and vapor pressure deficit (VPD); *LSTM_annual_* = *LSTM* model but the Landsat time series for each band were replaced by their annual mean, while using the actual values of T_*air*_, P, Rg, and VPD, RF = Random Forest model using the actual values of the Landsat time series and climate data.

	LSTM	LSTM_*perm*_	LSTM_*msc*_	LSTM_*annual*_	RF
Temporal feature extraction/Memory effects	**X**	**X**			**X**
Vegetation interannual seasonal variation	**X**		**X**		
Vegetation interannual variability	**X**			**X**	
Comparision to non-dynamic method	**X**				**X**

Comparing the *LSTM* with the *LSTM_msc_* model set-ups served to assess the importance of including and extracting information on interannual seasonal variation of vegetation to calculate NEE for each forest site across the globe. Contrasting the *LSTM_annual_* with the *LSTM* reflects lost in model fitness by not including the information contained in the monthly mean seasonal cycle of vegetation. The differences between the results from the *LSTM* and the *LSTM_perm_* as well as between the *LSTM* and the *RF* aimed to test the effects of extracting realistic temporal dependencies in the observations for predicting net CO_2_ fluxes. The *RF* set-up also provided a comparison to commonly used data-driven statistical modeling approaches for NEE estimates [62, 63] (Table 1). The predictive variables used in the different model set-ups are listed in Table 2.

**Table 2:** List of predictors used in the different model set-ups. T_*air*_ = air temperature, P = precipitation, Rg = global radiation, and VPD = vapor pressure deficit. *LSTM* = LSTM model using the full depth of the Landsat time series and climate data; *LSTM_perm_* = *LSTM* model but the temporal patterns of both the predictive and the target variables were randomly permuted while instantaneous relationships between predictive and target variables were kept; *LSTM_msc_* = *LSTM* model but the Landsat time series for each band were replaced by their mean seasonal cycle, while using the actual values of air temperature (T_*air*_), precipitation (P), global radiation (Rg), and vapor pressure deficit (VPD); *LSTM_annual_* = *LSTM* model but the Landsat time series for each band were replaced by their annual mean, while using the actual values of T_*air*_, P, Rg, and VPD, RF = Random Forest model using the actual values of the Landsat time series and climate data.

Model set-up	Predictors
LSTM	7 Landsat bands + T_*air*_ + P + Rg + VPD
LSTM_*perm*_	permuted [7 Landsat bands + T_*air*_ + P + Rg + VPD]
LSTM_*msc*_	7 MSC Landsat bands + T_*air*_ + P + Rg + VPD
LSTM_*annual*_	7 annual mean Landsat bands + T_*air*_ + P + Rg + VPD
RF	actual values of 7 Landsat bands + T_*air*_ + P + Rg +e VPD

#### Model training and evaluation

The performance of each model set-up was evaluated by directly comparing model estimates with observed values of NEE for each site. These statistical models were evaluated using a 10-fold cross-validation strategy in which entire sites were assigned to each fold [63]. The training of each model set-up was done using data from n_*fold*_-1, while predictions were made for the remaining fold, ensuring that the validation data were independent of the training data. The statistics used to assess the capability of the statistical models to estimate NEE were the coefficient of determination (R^2^), the NSE, the root mean squared error (RMSE), and the mean absolute error (MAE) [64]. The predictive capacity of the different algorithms was assessed for the seasonal cycle, the seasonal anomalies, the interannual anomalies, and the across-site variability. The seasonal anomalies were computed as the difference between the monthly NEE estimates of a considered month and those of the same month averaged over the observation period for each site. The interannual anomalies were computed as the difference between the annual NEE estimates of a considered year and the annual averaged over the entire observation period for each site. Both seasonal and interannual anomalies were calculated only for the sites with at least three years of complete observations after the data quality check. Across scales, the statistical models were trained using monthly time-series and the estimates were further aggregated to the corresponding scales, i.e. seasonal cycle, seasonal and interannual anomalies, and across-site.

Results were analyzed on the global dataset as well as according to PFT, bioclimatic and, forest age classes. PFT and climate classifications were found in the ancillary data files provided by the La Thuile or the FLUXNET2015 datasets https://fluxnet.fluxdata.org. Forest age data were derived from a published dataset [65]. Forest age estimates were aggregated in six classes: 0-10 years (n = 7 sites), 10-20 years (n = 8 sites), 20-50 years (n = 14 sites), 50-100 (n = 27 sites), 100-150 (n = 14 sites), and 150-≥300 years (n = 15 sites).

## Results and discussion

### Performance of the Recurrent Neural Networks

**Fig 4:**
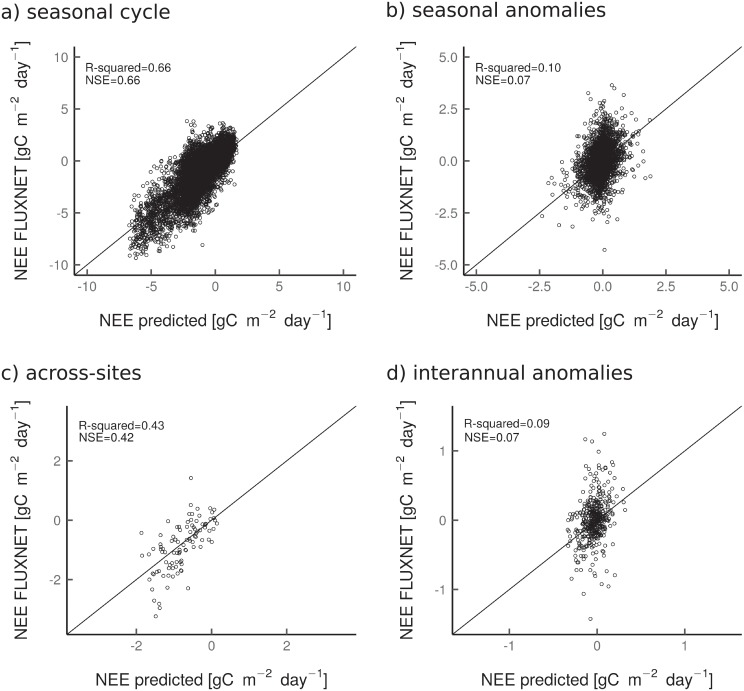
Scatterplots of observed data by eddy-covariance and the LSTM modeled fluxes for the seasonal cycle (Fig 4a), seasonal anomalies (Fig 4b), across-site variability (Fig 4c), and interannual anomalies (Fig 4d). The modeled estimates are derived from the mean ensemble of the 50 model runs.

The proposed approach was generally able to capture the seasonal cycle well for *LSTM* set-ups (NSE = 0.66), but had moderate to poor predictive capacity to explain across-site variability (NSE = 0.42), monthly anomalies (NSE = 0.07) or interannual anomalies (NSE = 0.07) (Fig 4). However, the proposed approach achieved comparable predictive capacity than the most recent NEE estimates based on FLUXNET data across scales [62, 63].

Such dynamic statistical modeling approach (i.e. LSTM) was expected to achieve a better performance for predicting anomalies compared to [62] and [63] analysis. In fact, LSTMs are theoretically able to automatically learn informative features [66, 67], and as such could capture interannual and seasonal fluctuations in the remote sensing and climate data related to specific ecosystem impact events (e.g. anthropogenic disturbances, seasonal droughts). However, this appeared not to be the case. We assume that this could be due to:

1. the fact that anomaly signals were relatively small (Fig 4b and 4d) compared to the low signal-to-noise ratio in the remote sensing data because of atmospheric contamination;
2. the non-availability of complete Landsat time series, and necessary gapfilling step;
3. the fact that the training of the statistical models was performed at monthly scale and not at daily scale due to the temporal resolution of the Landsat data. Signatures of extreme events are likely more apparent at daily time scale, therefore one could argue that the temporal scale used in the presented study is not appropriate to capture well the anomalies in the signals;
4. the limitations associated with the remote sensing signals in providing all necessary information regarding vegetation structure and growth trajectory, while being insensitive to C decomposition dynamics;
5. the fact that few disturbances events were observed during the observational period compared to undisturbed sites or to sites where disturbances occurred a long time ago in which no spectral recovery signals were captured during the training procedure (i.e. only 10% of the observations in recently disturbed forests); and
6. the lack of information related to the spatial context (e.g. landscape patchiness, fractal dimension) that could translate the development stage.

All these factors could suggest that few anomaly signals are captured during the training process of the proposed approach. Furthermore, we cannot ignore the fact that there is a lack of relevant information on the predictors used in this study to predict NEE variability across-scales. For instance, extracting temporal variation in the Landsat data could have been a good proxy for the age effects on NEE among sites, but this was not the case here (as discussed further), likely due to missing information in the input variables for the heterotrophic respiration component of NEE. The mismatch between the observed and predicted NEE at interannual scales could also be related to the fact that the training procedure does not learn site-specific characteristics due to the implemented cross-validation set-up (i.e. entire sites out cross-validation) [40], therefore limiting the capacity of a statistical model to predict NEE interannual anomalies accurately. Furthermore, the cost function was performed on the monthly observations during the training/evaluation procedure, which can potentially limit the capacity of the presented approach for calculating NEE signals realistically at annual scales. In addition, there are few very young sites (< 20 years old) or sites where disturbances occurred during the Landsat record in the training set, which can limit the ability of the proposed approach to have good predictive capacity in young recently disturbed sites. Another source of uncertainty is related to the mismatch between flux measurements and the Landsat time series cutouts around each flux tower. To overcome the latter issue, integration of footprint analysis could help to better describe the origin of the fluxes within the Landsat time series cutouts.

Differences in predictive capacity were apparent for different PFTs and climate levels (Table 3, S2 and S3 Tables). The NSE for different PFTs and climate regions at the seasonal scale ranged from 0.42 (i.e. evergreen forests) to 0.82 (i.e. deciduous forests), and from -0.0006 for the tropical forests to 0.68 for both temperate and boreal forests. The fact that the LSTM showed poor agreement with observations in the tropics can be explained by the very small signal in the input data due to the lack of seasonal variation in terms of reflective, thermal, and moisture properties [32]. In addition, the Landsat data tend to be very sparse in the tropics due to frequent cloud coverage, leading to a high fraction of gap-filled data, thus a potentially poor representation of the seasonal vegetation variation. The properties of the Landsat data might also not be suitable to characterize seasonality in the tropics, therefore other remote sensing products related to leaf development and demography (e.g. Multi-Angle Implementation of Atmospheric Correction data product) [68] could be explored. However, such products were not tested given their relatively short time series, although this shortcoming may be overcome in the future. EC flux data also have their own limitations in the tropics, not only due to sparse spatial coverage but also due to large gaps in data related to frequent rain events and severe issues with the night-time fluxes due to low wind speed and tall canopies. The *LSTM* was able to well predict NEE across-sites in evergreen forests (NSE = 0.43), while it showed poor agreement with observations in tropical regions (NSE = -0.15). One could assume that the low number of tropical sites currently available in this dataset (n = 16 and n = 7) at the seasonal scale and across-sites, respectively) might limit an LSTM to predict spatial NEE variabilities in such an ecosystem and also lead to a systematically higher uncertainty across-scales compared to other PFTs (Table 3).

**Table 3:** Nash-Sutcliffe modeling efficiency of the *LSTM* setup per vegetation type and climate region from the ensemble mean ±sd estimate of the 50 runs. Statistics for the anomalies were not calculated in the arid and tropical climate (i.e. NA) because there was no site with at least 3 years of complete data after data quality control. Savanna vegetation type includes both savanna and woody savanna sites.

	Seasonal cycle	Seasonal anomalies	Across-sites	Interannual anomalies
Deciduous forest	**0.82** ±0.01	**0.16** ±0.03	**0.26** ±0.07	**0.17** ±0.04
Evergreen forest	**0.42** ±0.02	**0.03** ±0.02	**0.43** ±0.06	**0.008** ±0.04
Mixed forest	**0.63** ±0.03	**0.01** ±0.02	**0.40** ±0.19	**0.08** ±0.04
Savanna	**0.55** ±0.02	**0.03** ±0.02	**0.29** ±0.48	**0.37** ±0.07
Arid	**0.47** ±0.04	NA	**0.15** ±0.75	NA
Boreal	**0.68** ±0.01	**0.14** ±0.03	**0.33** ±0.07	**-0.04** ±0.05
Temperate	**0.68** ±0.01	**0.04** ±0.02	**0.29** ±0.07	**0.09** ±0.03
Tropical	**-0.0006** ±0.12	NA	**-0.15** ±0.27	NA

### Comparison of the different model set-ups

A comparison of the model performance was done between the different LSTM networks along with the non-dynamic statistical RF model (Table 4, S4, S5 and S6 Tables). In general, the performance metrics across the model set-ups differed. All model set-ups were capable of well predicting the seasonal cycle, with the *LSTM* achieving particularly better model fitness and lower errors (NSE = 0.66, RMSE = 1.12, and MAE = 0.81). Similarly, *LSTM* depicted better agreement between observations and predictions across-sites (NSE = 0.42, RMSE = 0.63, and MAE = 0.48). However, none of the presented model set-ups were able to successfully predict the anomaly signals, with *LSTM* having rather similar performance and level of errors than the other model set-ups for both seasonal (NSE = 0.07, RMSE = 0.61, and MAE = 0.31) and interannual anomalies (NSE = 0.07, RMSE = 0.31, and MAE = 0.22). Still, this results supported the importance of accounting for interannual and seasonal fluctuations of climate and vegetation to estimate net CO_2_ fluxes, in particular at the seasonal scale and across-sites. This was evidenced by *LSTM_msc_*, *LSTM_perm_*, *LSTM_annual_*, and the *RF* model set-ups, which depicted lower predictive capacities and higher errors than the original *LSTM*. However, these comparisons were done for the entire FLUXNET dataset, but the effects of memory were substantially different across-sites (S4 Fig).

**Table 4:** Nash-Sutcliffe modeling efficiency of the proposed approach against the other model set-ups from the ensemble mean ±sd estimate of the 50 model runs. *LSTM* = LSTM model using the full depth of the Landsat time series and climate data; *LSTM_perm_* = *LSTM* model but the temporal patterns of both the predictive and the target variables were randomly permuted while instantaneous relationships between predictive and target variables were kept; *LSTM_msc_* = *LSTM* model but the Landsat time series for each band were replaced by their mean seasonal cycle, while using the actual values of air temperature (T_*air*_), precipitation (P), global radiation (Rg), and vapor pressure deficit (VPD); *LSTM_annual_* = *LSTM* model but the Landsat time series for each band were replaced by their annual mean, while using the actual values of T_*air*_, P, Rg, and VPD, RF = Random Forest model using the actual values of the Landsat time series and climate data.

	Seasonal cycle	Seasonal anomalies	Across-sites	Interannual anomalies
LSTM	**0.66** ±0.01	**0.07** ±0.01	**0.42** ±0.05	**0.07** ±0.03
LSTM_*msc*_	**0.64** ±0.009	**0.05** ±0.008	**0.39** ±0.04	**0.02** ±0.01
LSTM_*annual*_	**0.59** ±0.02	**0.06** ±0.03	**0.36** ±0.05	**0.04** ±0.05
LSTM_*perm*_	**0.61** ±0.01	**0.008** ±0.02	**0.38** ±0.05	**0.08** ±0.02
RF	**0.58** ±0.00004	**-0.30** ±0.0006	**0.38** ±0.0002	**-0.04** ±0.0007

**Fig 5:**
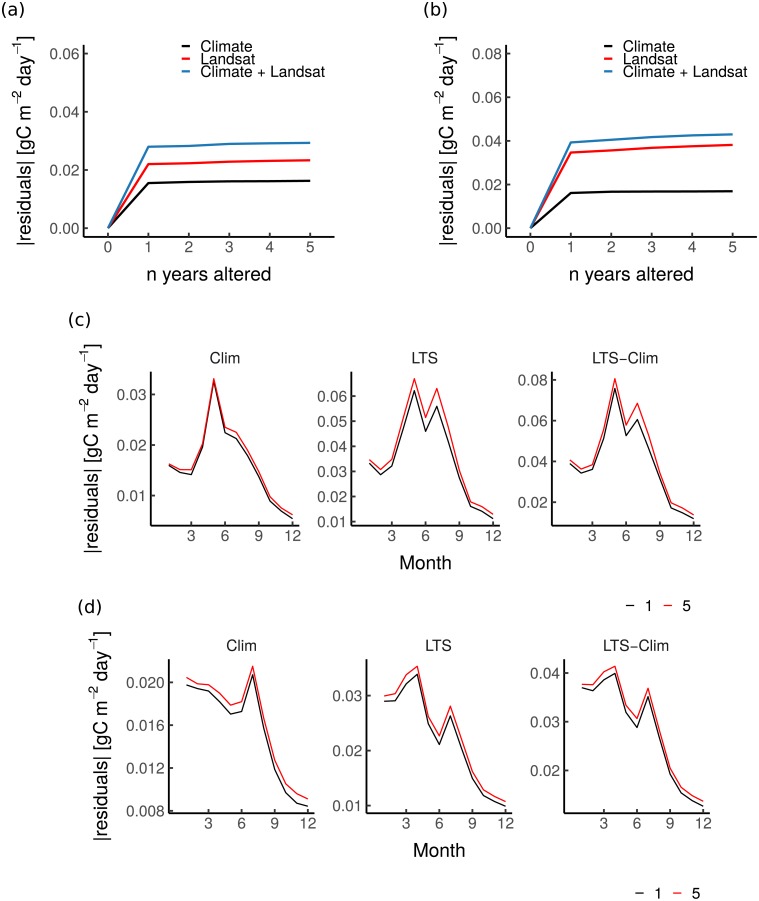
Effects on predicting monthly NEE by altering n year in the predictors for deciduous and evergreen forests. Average of the absolute residuals calculated between predicted monthly NEE with 0 year altered in the predictors against predicted monthly NEE with year_*i*−*n*_ altered in the predictors for deciduous and evergreen forests (Fig 5a and b, respectively). The absolute residuals for the mean seasonal cycle were also reported (Fig 5c and d for deciduous and evergreen forests, respectively). “1 year” means that only the last year was altered, “2 years” means that the last two years were altered, and so on. Months for the sites located in the Southern hemisphere have been adjusted to match the seasonal cycle of the sites in the Northern hemisphere.

The fact that the *LSTM* network exploits the history of the predictor variables could explain its overall better results in predicting CO_2_ fluxes compared to other model set-ups, despite the differences being marginal. The CO_2_ fluxes are not only linearly related to the instantaneous reflectance and meteorological conditions but also associated with the climate and vegetation dynamics several months to years prior [6], which may affect non-observed ecosystem states with direct consequences to C fluxes. To investigate this, an additional simulation experiment was conducted to understand how many years, before predicting a specific year, the proposed approach (i.e. *LSTM*) uses to achieve a better model performance (Fig 5). The *LSTM* model trained before was used, but during the prediction, the actual values for predictors in year_*i*−*n*_ (where n is a number of years ranging from one to five) were replaced by their MSC when predicting year_*i*_. Hence, the interannual variations and seasonal deviations of year_*i*−*n*_ were not included in the predictions of the LSTMs when calculating NEE for year_*i*_. For both deciduous and evergreen forests, there was a consistent increase in the mean absolute residuals from 0 to 1 years of altered forcings, while there were no substantial changes when the number of years since alteration was ≥ 1 year (Fig 5a and 5b). It is also interesting to see that altering only climate predictors has less of an effect on the deviations from the NEE estimates, compared to the other two scenarios. For deciduous forests, capturing information from the current and the previous years results in the highest differences in NEE estimates mainly during the growing season (April to September) (Fig 5c). On the other hand, altering the Landsat and climate time series of the previous years seemed to mainly have the highest effects on the predicted NEE from January to August-September. Overall, the magnitudes in the errors are substantially higher for deciduous forests. Note that these findings do not mean that only previous-year climate and vegetation memory effects are important for improving NEE estimates but indicate that their significance in the proposed approach diminishes to further improve its predictive capacity.

**Fig 6:**
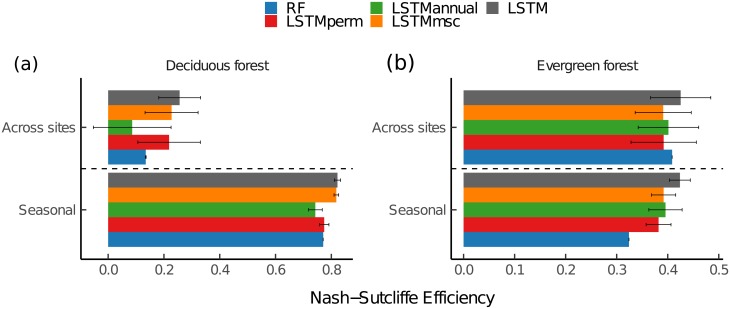
Nash-Sutcliffe modeling efficiency comparison between the proposed LSTM-based models and the other model set-ups for (a) deciduous and (b) evergreen forests. Nash-Sutcliffe modeling efficiency values have been calculated based on the mean ensemble ±sd of the 50 model runs. *LSTM* = LSTM model using the full depth of the Landsat time series and climate data; *LSTM_perm_* = *LSTM* model but the temporal patterns of both the predictive and the target variables were randomly permuted while instantaneous relationships between predictive and target variables were kept; *LSTM_msc_* = *LSTM* model but the Landsat time series for each band were replaced by their mean seasonal cycle, while using the actual values of air temperature (T_*air*_), precipitation (P), global radiation (Rg), and vapor pressure deficit (VPD); *LSTM_annual_* = *LSTM* model but the Landsat time series for each band were replaced by their annual mean, while using the actual values of T_*air*_, P, Rg, and VPD, RF = Random Forest model using the actual values of the Landsat time series and climate data.

This study confirms that changes in historical climate and vegetation dynamics play a moderate role in shaping the temporal variability of ecosystem CO_2_ fluxes, particularly at the seasonal scale (i.e. around 8% difference in model efficiency between *LSTM* and *LSTM_perm_*) and across-sites (i.e. 10% difference in model efficiency between *LSTM* and *LSTM_perm_*) (Table 4 and Fig 5). However, these findings differ markedly between forest types (Figs 5 and 6). For instance, NEE estimates calculated by *LSTM* and *LSTM_msc_* for deciduous forests are rather similar at the seasonal scale, suggesting that the interannual variation information carried by the remote sensing data does not help to achieve better performance capacity in predicting NEE at seasonal scale in such an ecosystem, while the interannual variation in climate is still considered. On the other hand, the highest modeling efficiency was achieved for evergreen forests using the *LSTM* model, suggesting that both interannual and seasonal fluctuations in vegetation are important drivers of NEE variabilities at the seasonal scale.

**Fig 7:**
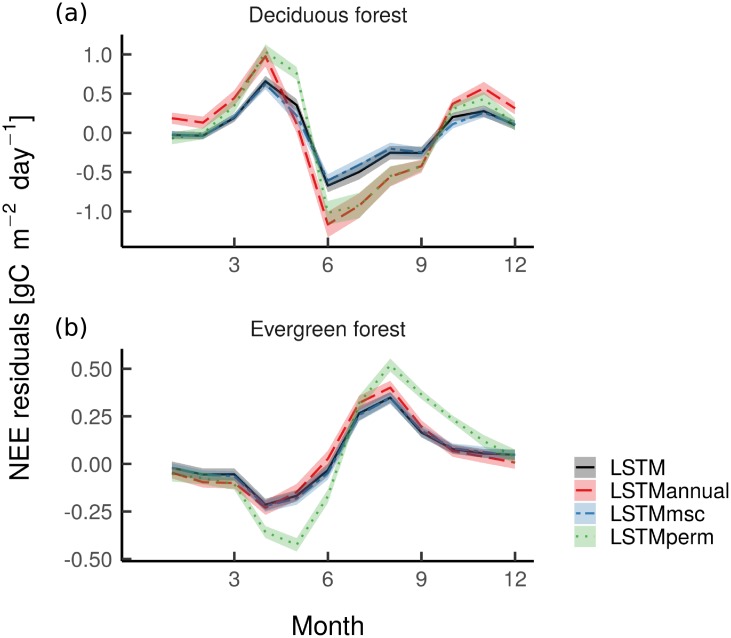
Mean seasonal variation of NEE residuals for *LSTM*, *LSTM_perm_*, *LSTM_msc_*, and *LSTM_annual_* models for (a) deciduous and (b) evergreen forests. NEE residuals = [NEE observed_*i*,*j*_ − mean(NEE observed_*i*_)] − [NEE predicted_*i*,*j*_ − mean(NEE predicted_*i*_)], where i is a unique Fluxnet site and j is a monthly observation. Residual estimates have been calculated based on the mean ensemble ±sd of the 50 model runs. *LSTM* = LSTM model using the full depth of the Landsat time series and climate data; *LSTM_perm_* = *LSTM* model but the temporal patterns of both the predictive and the target variables were randomly permuted while instantaneous relationships between predictive and target variables were kept; *LSTM_msc_* = *LSTM* model but the Landsat time series for each band were replaced by their mean seasonal cycle, while using the actual values of air temperature (T_*air*_), precipitation (P), global radiation (Rg), and vapor pressure deficit (VPD); *LSTM_annual_* = *LSTM* model but the Landsat time series for each band were replaced by their annual mean, while using the actual values of T_*air*_, P, Rg, and VPD, RF = Random Forest model using the actual values of the Landsat time series and climate data. Months for the sites located in the Southern hemisphere have been adjusted to match the seasonal cycle of the sites in the Northern hemisphere.

One outstanding result of this analysis is the importance of memory effect at the seasonal scale (Table 4 and Fig 6). Such finding can be better explored using the NEE mean seasonal variation residuals for deciduous and evergreen forests (Fig 7). For deciduous and evergreen forests, it is important to extract realistic temporal vegetation and climate information when predicting NEE as the *LSTM_perm_* model depicts the highest overall error in the residual seasonal patterns compared to the other model set-ups. For deciduous forests, both the onset and the peak of the growing season were better captured by *LSTM* and *LSTM_msc_* models (Fig 7a). This could suggest that the climatic conditions of the previous years (e.g. water limitations [33, 35], increased precipitation [34], or minimum air temperature during spring of the previous year [36]) not only control NEE seasonal patterns in deciduous forests but also mean seasonal vegetation fluctuations. It is therefore probable that seasonal leaf physiology due to leaf aging also drives the residual seasonal patterns [69]. The *LSTM_annual_* model set-up revealed that capturing interannual variations in vegetation activities does not help in representing NEE estimates at the seasonal scale. However, all the model set-ups showed rather similar errors when representing the senescence phase in deciduous forests, suggesting that the processes that control these dynamics are not expressed in any of the observational datasets used here. Interestingly, *LSTM*, *LSTM_msc_*, and *LSTM_annual_* model set-ups depicted relatively similar errors over the course of the growing season for evergreen forests (Fig 7b). This means that both the current climate conditions and the ones of the previous months or years control NEE seasonal cycle in such an ecosystem. These findings confirmed the existence of different ecosystem type-specific memory or lagged effects [33].

**Fig 8:**
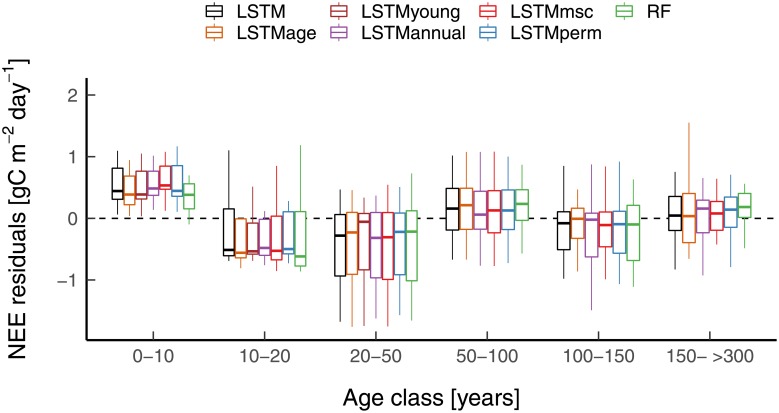
Model residuals per age class for *LSTM*, *LSTM_perm_*, *LSTM_msc_*, *LSTM_annual_*, and *RF* models based on site-average NEE. *LSTM* = LSTM model using the full depth of the Landsat time series and climate data; *LSTM_perm_* = *LSTM* model but the temporal patterns of both the predictive and the target variables were randomly permuted while instantaneous relationships between predictive and target variables were kept; *LSTM_msc_* = *LSTM* model but the Landsat time series for each band were replaced by their mean seasonal cycle, while using the actual values of air temperature (T_*air*_), precipitation (P), global radiation (Rg), and vapor pressure deficit (VPD); *LSTM_annual_* = *LSTM* model but the Landsat time series for each band were replaced by their annual mean, while using the actual values of T_*air*_, P, Rg, and VPD, RF = Random Forest model using the actual values of the Landsat time series and climate data.; *LSTM_age_* = *LSTM* + forest age as a predictive variable; *LSTM_young_* = *LSTM* only trained with forests younger than 40 years.

The *LSTM* model set-up outperformed the other models (i.e. *LSTM_perm_*, *LSTM_msc_*, *LSTM_annual_*, and *RF*) across sites, suggesting that it is able to better capture the complexity of the relation between past dynamics and current functions of the forests across-space. One hypothesis could be that net CO_2_ fluxes in recently disturbed forests are better predicted with a method that captures disturbance regimes. However, this hypothesis could not be confirmed since: (1) the *LSTM* model set-up did not outperform the other model set-ups for young forests (i.e. 0-20 years old) and for recently disturbed forests (Fig 8); and (2) training an LSTM adding forest age as predictor or training it only for young forests (forest age < 40 years) did not correct for the bias in young forests (Fig 8). However, it is not possible to be conclusive on the ability of the LSTMs to predict young sites since: (1) there was only a small sample of young forests and recently disturbed sites in this dataset; and that methodologically (2) no *in-situ* proxies for productivity were used in the analysis (e.g. related gross primary productivity); and (3) the LSTMs were trained with monthly observation. Therefore, it is very likely that the better performance of the *LSTM* model set-up compared to the other model set-ups at the seasonal cycle could explain its overall better capacity in explaining NEE spatial variation (e.g. spring NEE accounts for most of the annual NEE in the temperate deciduous forests [36]).

## Conclusion

In this study, we present a novel framework for assessing the potential of the memory effects of climate and vegetation on forests’ NEE using the Landsat satellite imagery and *in-situ* eddy covariance observations. The results presented here for the whole FLUXNET dataset reveal a variable memory effect on NEE across-scales, but that is mainly apparent at the seasonal scale and across-sites. We also find that the effects of memory vary between FLUXNET sites suggesting site-specific memory effects. Although instantaneous observations of the contemporaneous vegetation states may already carry information from the past, current analysis suggests that extracting antecedent observations of vegetation and climate are beneficial for estimating NEE more realistically (the difference between *LSTM* and *LSTM_perm_*, as well as between *LSTM* and *RF*). Such effects can emerge from the information contained in the course of the seasonal cycle or from the effects of interannual variation on NEE. However, the close agreement between *LSTM* and *LSTM_msc_* suggests that either the effect is smeared out by the impact of instantaneous climate on NEE or the interannual variation’s memory effect in NEE is implicitly captured by this approach. The results are contingent on the length of observations and few recently disturbed forests but do emphasize the possibility of dynamic statistical methods that include memory effects to better estimate the contribution of forest ecosystems in the global terrestrial C cycle, hence for further improving statistically-based prediction methods.

## Supporting information

S1 Table List of sites used in this study. DBF = Deciduous broadleaf forest, DNF = deciduous needleleaf forest, EBF = evergreen broadleaf forest, ENF = evergreen needleleaf forest, MF = mixed forest, WSA = woody savanna, and SAV = savanna.
(PDF)

S2 Table RMSE of the *LSTM* setup per PFT and climate region from the ensemble mean mean ±sd estimate of the 50 runs. Statistics for the anomalies were not calculated in the arid and tropical climate (i.e. NA) because there was no site with at least 2 years of complete data after data quality control.
(PDF)

S3 Table MAE of the *LSTM* setup per PFT and climate region from the ensemble mean mean ±sd estimate of the 50 runs. Statistics for the anomalies were not calculated in the arid and tropical climate (i.e. NA) because there was no site with at least 2 years of complete data after data quality control.
(PDF)

S4 Table Coefficient of determination of the proposed approach against the other model set-ups from the ensemble mean mean ±sd estimate of the 50 runs. *LSTM* = LSTM model using the full depth of the Landsat time series and climate data; *LSTM_perm_* = *LSTM* model but the temporal patterns of both the predictive and the target variables were randomly permuted while instantaneous relationships between predictive and target variables were kept; *LSTM_msc_* = *LSTM* model but the Landsat time series for each band were replaced by their mean seasonal cycle, while using the actual values of air temperature (T_*air*_), precipitation (P), global radiation (Rg), and vapor pressure deficit (VPD); *LSTM_annual_* = *LSTM* model but the Landsat time series for each band were replaced by their annual mean, while using the actual values of T_*air*_, P, Rg, and VPD, RF = Random Forest model using the actual values of the Landsat time series and climate data.
(PDF)

S5 Table RMSE of the proposed approach against the other model set-ups from the ensemble mean mean ±sd estimate of the 50 runs. *LSTM* = LSTM model using the full depth of the Landsat time series and climate data; *LSTM_perm_* = *LSTM* model but the temporal patterns of both the predictive and the target variables were randomly permuted while instantaneous relationships between predictive and target variables were kept; *LSTM_msc_* = *LSTM* model but the Landsat time series for each band were replaced by their mean seasonal cycle, while using the actual values of air temperature (T_*air*_), precipitation (P), global radiation (Rg), and vapor pressure deficit (VPD); *LSTM_annual_* = *LSTM* model but the Landsat time series for each band were replaced by their annual mean, while using the actual values of T_*air*_, P, Rg, and VPD, RF = Random Forest model using the actual values of the Landsat time series and climate data.
(PDF)

S6 Table MAE of the proposed approach against the other model set-ups from the ensemble mean mean ±sd estimate of the 50 runs. *LSTM* = LSTM model using the full depth of the Landsat time series and climate data; *LSTM_perm_* = *LSTM* model but the temporal patterns of both the predictive and the target variables were randomly permuted while instantaneous relationships between predictive and target variables were kept; *LSTM_msc_* = *LSTM* model but the Landsat time series for each band were replaced by their mean seasonal cycle, while using the actual values of air temperature (T_*air*_), precipitation (P), global radiation (Rg), and vapor pressure deficit (VPD); *LSTM_annual_* = *LSTM* model but the Landsat time series for each band were replaced by their annual mean, while using the actual values of T_*air*_, P, Rg, and VPD, RF = Random Forest model using the actual values of the Landsat time series and climate data.
(PDF)

S1 Fig Performance of the gap-filling procedure of each Landsat band using a Random Forest model and the MODIS bands as predictive variables. The model was trained on 70% of the data and evaluated on 30% of the left out data. nir = near-infrared, swir1 = shortwave infrared 1, swir2 = shortwave infrared 2, and tir = thermal infrared.
(PDF)

S2 Fig Performance of the gap-filling procedure of each Landsat band using a Random Forest model and climate variables (i.e. T_*air*_, Precip, Rg, VPD, rpot), PFT, month of the year, and latitude as predictive variables. The model was trained on 70% of the data and evaluated on 30% of the left out data. nir = near-infrared, swir1 = shortwave infrared 1, swir2 = shortwave infrared 2, and tir = thermal infrared.
(PDF)

S3 Fig Performance of the gap-filling procedure for the differtent climate variables. Assessment of the gap-filling procedure was done for T_*air*_, Precip, Rg, and VPD. For T_*air*_, Rg, and VPD, the Nash-Sutcliffe efficiency (NSE) is reported, while the root mean squared error (RMSE) is reported for Precip.
(PDF)

S4 Fig Scatterplots of the coefficient of determination of the proposed approach against the other model set-ups at site level. The coefficient of determination was computed using monthly observed and predicted NEE estimates for each site. Each point represents one site and only the sites with at least one complete year of good quality data (n site = 81) are shown.
(PDF)
