# Correction: Memory effects of climate and vegetation affecting net ecosystem CO_2_ fluxes in global forests

**DOI:** 10.1371/journal.pone.0213467

**Published:** 2019-02-28

**Authors:** The PLOS ONE Staff

Fig 1 is incorrect. The publisher apologizes for the error. The authors have provided a corrected version here.

**Fig 1:**
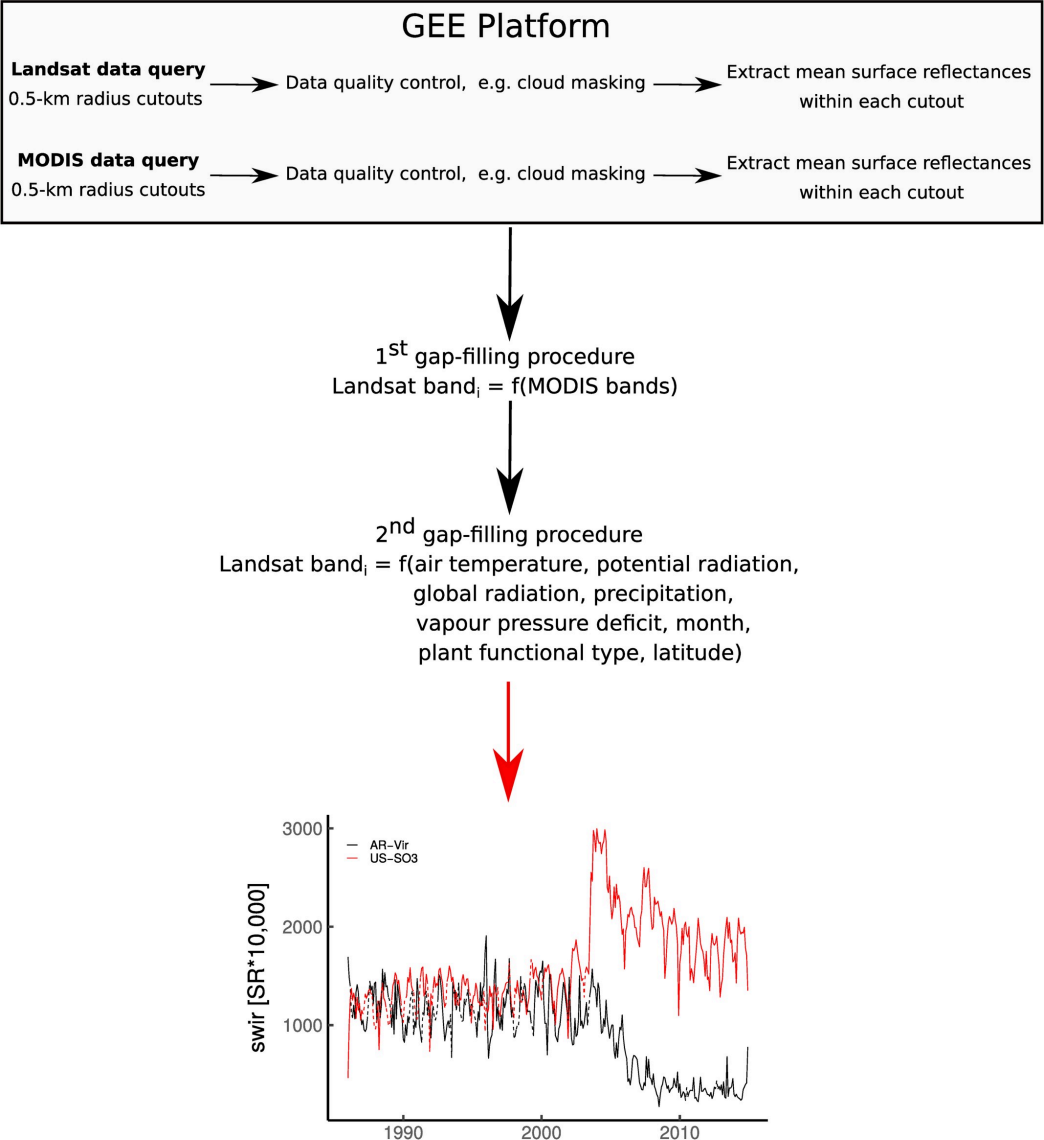
Flowchart of the Landsat data extraction and post-processing. SWIR = Shortwave Infrared. SR = Surface Reflectance. Monthly temporal gap-filled Landsat time series from 1982 to 2015 of the shortwave Infrared band are shown for AR-Vir and US-SO3 sites where, respectively, afforestation-reforestation and fire followed by a regrowth were reported in 2003. The solid and the dashed lines depict the real observations and the gap-filled data, respectively.

## References

[1] Besnard S, Carvalhais N, Arain MA, Black A, Brede B, Buchmann N, et al (2019) Memory effects of climate and vegetation affecting net ecosystem CO_2_ fluxes in global forests. PLoS ONE 14(2): e0211510 10.1371/journal.pone.0211510 30726269 PMC6364965

