# Supplementary material for: Memory effects of climate and vegetation affecting net ecosystem CO2 fluxes in global forests
Source: PLoS One. 2019 Feb 6;14(2):e0211510. doi: 10.1371/journal.pone.0211510 (PMC6364965; doi:10.1371/journal.pone.0211510)
Supplement: S1 Table — DBF = Deciduous broadleaf forest, DNF = deciduous needleleaf forest, EBF = evergreen broadleaf forest, ENF = evergreen needleleaf forest, MF = mixed forest, WSA = woody savanna, and SAV = savanna. (PDF) [file pone.0211510.s001.pdf]

S1 Table. List of sites used in this study. DBF = Deciduous broadleaf forest, DNF = deciduous needleleaf forest, EBF = evergreen broadleaf forest, ENF = evergreen needleleaf forest, MF = mixed forest, WSA = woody savanna, and SAV = savanna. The PFT and climate classifications used here are coming from the ancillary data files provided by the La Thuile or the FLUXNET2015 datasets (`https://fluxnet.fluxdata.org`).

	Site ID	Lat [°N]	Long [°E]	Climate type	Vegetation type	Forest age	References
1	AR-SLu	-33.4648	-66.4598	Temperate	MF	50	-
2	AR-Vir	-28.2395	-56.1886	Temperate	ENF	8	-
3	AU-Ade	-13.0769	131.1178	Tropical	WSA	NA	-
4	AU-ASM	-22.2830	133.2490	Arid	ENF	NA	-
5	AU-Cpr	-34.0021	140.5891	Arid	SAV	NA	-
6	AU-Cum	-33.6133	150.7225	Temperate	EBF	>300	-
7	AU-DaS	-14.1593	131.3881	Tropical	SAV	NA	-
8	AU-Dry	-15.2588	132.3706	Tropical	SAV	NA	-
9	AU-Gin	-31.3764	115.7138	Temperate	WSA	NA	-
10	AU-GWW	-30.1913	120.6541	Arid	SAV	NA	-
11	AU-How	-12.4943	131.1523	Tropical	WSA	110	-
12	AU-Lox	-34.4704	140.6551	Arid	DBF	NA	-
13	AU-RDF	-14.5636	132.4776	Tropical	WSA	NA	-
14	AU-Rob	-17.1175	145.6301	Temperate	EBF	198	-
15	AU-Tum	-35.6566	148.1517	Temperate	EBF	83	[27]
16	AU-Wac	-37.4259	145.1878	Temperate	EBF	>300	-
17	AU-Whr	-36.6732	145.0294	Temperate	EBF	NA	-
18	AU-Wom	-37.4222	144.0944	Temperate	EBF	32	-
19	BE-Bra	51.3092	4.5206	Temperate	MF	78	[21]
20	BE-Jal	50.5639	6.0733	Temperate	MF	8	-
21	BE-Vie	50.3051	5.9981	Temperate	MF	94	[4]
22	BR-Ban	-9.8244	-50.1591	Tropical	EBF	>300	[19]
23	BR-Cax	-1.7197	-51.4590	Tropical	EBF	96	[14]
24	BR-Ji2	-10.0832	-61.9309	Tropical	EBF	>300	[88]

Continued on next page

	Site ID	Lat [°N]	Long [°E]	Climate type	Vegetation type	Forest age	References
25	BR-Ma2	-2.6091	-60.2093	Tropical	EBF	>300	[3]
26	BR-Sa1	-2.8567	-54.9589	Tropical	EBF	>300	[74]
27	BR-Sa3	-3.0180	-54.9714	Tropical	EBF	>300	[74]
28	BR-Sp1	-21.6195	-47.6499	Tropical	WSA	NA	-
29	BW-Ma1	-19.9155	23.5605	Arid	WSA	NA	-
30	CA-Ca1	49.8672	-125.3340	Temperate	ENF	60	[60]
31	CA-Ca2	49.8705	-125.2910	Temperate	ENF	3	[41]
32	CA-Ca3	49.5346	-124.9000	Temperate	ENF	16	[44]
33	CA-Gro	48.2167	-82.1556	Boreal	MF	78	[66]
34	CA-Man	55.8796	-98.4808	Boreal	ENF	161	[46]
35	CA-NS1	55.8792	-98.4839	Boreal	ENF	154	[32]
36	CA-NS2	55.9058	-98.5247	Boreal	ENF	73	[32]
37	CA-NS3	55.9117	-98.3822	Boreal	ENF	39	[32]
38	CA-NS4	55.9144	-98.3806	Boreal	ENF	39	[32]
39	CA-NS5	55.8631	-98.4850	Boreal	ENF	23	[32]
40	CA-Oas	53.6289	-106.1978	Boreal	DBF	80	[10]
41	CA-Obs	53.9872	-105.1178	Boreal	ENF	112	[43]
42	CA-Ojp	53.9163	-104.6920	Boreal	ENF	88	[5]
43	CA-Qcu	49.2671	-74.0365	Boreal	ENF	4	[28]
44	CA-Qfo	49.6925	-74.3421	Boreal	ENF	102	[7]
45	CA-SF1	54.4850	-105.8176	Boreal	ENF	28	-
46	CA-SF2	54.2539	-105.8775	Boreal	ENF	14	-
47	CA-SJ1	53.9080	-104.6560	Boreal	ENF	10	[40]
48	CA-SJ2	53.9450	-104.6490	Boreal	ENF	2	[18]
49	CA-SJ3	53.8758	-104.6450	Boreal	ENF	30	[33]
50	CA-TP1	42.6609	-80.5595	Boreal	ENF	9	[2]
51	CA-TP2	42.7744	-80.4588	Boreal	ENF	73	[2]
52	CA-TP3	42.7068	-80.3483	Boreal	ENF	37	[2]
53	CA-TP4	42.7102	-80.3574	Boreal	ENF	70	[2]

Continued on next page

	Site ID	Lat [°N]	Long [°E]	Climate type	Vegetation type	Forest age	References
54	CA-TPD	42.6353	-80.5577	Boreal	DBF	98	[2]
55	CG-Hin	-4.6811	12.0036	Tropical	EBF	NA	-
56	CG-Kis	-4.7914	11.9822	Tropical	EBF	NA	-
57	CG-Tch	-4.2892	11.6564	Tropical	SAV	NA	-
58	CH-Dav	46.8153	9.8559	Temperate	ENF	222	[92]
59	CH-Lae	47.4781	8.3650	Temperate	MF	184	[26]
60	CN-Anh	33.0000	117.0000	Temperate	DBF	13	-
61	CN-Bed	39.5306	116.2520	Boreal	EBF	12	-
62	CN-Cha	42.4025	128.0958	Boreal	MF	>300	[37]
63	CN-Din	23.1733	112.5361	Temperate	EBF	96	[91]
64	CN-Hny	29.3100	112.5100	Temperate	DBF	2	-
65	CN-Ku1	40.5383	108.6940	Arid	EBF	5	-
66	CN-Qia	26.7414	115.0581	Temperate	ENF	19	-
67	CZ-BK1	49.5021	18.5369	Boreal	ENF	31	[69]
68	DE-Bay	50.1419	11.8669	Temperate	ENF	54	[85]
69	DE-Hai	51.0792	10.4530	Temperate	DBF	254	[47]
70	DE-Har	47.9344	7.6010	Temperate	ENF	42	[9]
71	DE-Lkb	49.0996	13.3047	Temperate	ENF	2	[51]
72	DE-Lnf	51.3282	10.3678	Temperate	DBF	117	-
73	DE-Meh	51.2753	10.6555	Temperate	MF	2	-
74	DE-Obe	50.7836	13.7196	Temperate	ENF	76	-
75	DE-Tha	50.9636	13.5669	Temperate	ENF	118	[8]
76	DE-Wet	50.4535	11.4575	Temperate	ENF	54	[1]
77	DK-Sor	55.4859	11.6446	Temperate	DBF	85	[67]
78	ES-ES1	39.3460	-0.3188	Temperate	ENF	116	[75]
79	ES-LMa	39.9415	-5.7734	Temperate	SAV	148	-
80	FI-Hyy	61.8475	24.2950	Boreal	ENF	46	[77]
81	FI-Let	60.6418	23.9597	Boreal	ENF	NA	-
82	FI-Sod	67.3619	26.6378	Boreal	ENF	161	[81]

Continued on next page

	Site ID	Lat [°N]	Long [°E]	Climate type	Vegetation type	Forest age	References
83	FR-Fon	48.4764	2.7801	Temperate	DBF	150	[56]
84	FR-Hes	48.6742	7.0646	Temperate	DBF	37	[34]
85	FR-LBr	44.7171	-0.7693	Temperate	ENF	34	[6]
86	FR-Pue	43.7414	3.5958	Temperate	EBF	64	[68]
87	GF-Guy	5.2788	-52.9249	Tropical	EBF	>300	[11]
88	GH-Ank	5.2685	-2.6942	Tropical	EBF	NA	-
89	IL-Yat	31.3450	35.0515	Arid	ENF	39	[35]
90	IS-Gun	63.8333	-20.2167	Temperate	DBF	7	-
91	IT-Bon	39.4778	16.5347	Temperate	ENF	36	-
92	IT-CA1	42.3804	12.0266	Temperate	DBF	NA	-
93	IT-CA3	42.3800	12.0222	Temperate	DBF	NA	-
94	IT-Col	41.8494	13.5881	Temperate	DBF	180	[84]
95	IT-Cp2	41.7043	12.3573	Temperate	EBF	63	-
96	IT-Cpz	41.7052	12.3761	Temperate	EBF	56	[82]
97	IT-Isp	45.8126	8.6336	Temperate	DBF	NA	-
98	IT-Lec	43.3046	11.2706	Temperate	EBF	NA	-
99	IT-LMa	45.5813	7.1546	Temperate	DBF	71	[54]
100	IT-Non	44.6898	11.0887	Temperate	MF	10	[62]
101	IT-PT1	45.2009	9.0610	Temperate	DBF	13	[57]
102	IT-Ren	46.5869	11.4337	Boreal	ENF	188	[59]
103	IT-Ro1	42.4081	11.9300	Temperate	DBF	10	[70]
104	IT-Ro2	42.3903	11.9209	Temperate	DBF	19	[80]
105	IT-SR2	43.7320	10.2910	Temperate	ENF	64	-
106	IT-SRo	43.7279	10.2844	Temperate	ENF	54	[15]
107	IT-Vig	45.3167	8.8500	Temperate	DBF	15	-
108	JP-MBF	44.3869	142.3186	Boreal	DBF	NA	-
109	JP-SMF	35.2617	137.0788	Temperate	MF	NA	-
110	JP-Tak	36.1462	137.4230	Boreal	DBF	72	[90]
111	JP-Tef	45.0563	142.1062	Boreal	MF	121	[78]

Continued on next page

	Site ID	Lat [°N]	Long [°E]	Climate type	Vegetation type	Forest age	References
112	JP-Tom	42.7395	141.5149	Boreal	MF	48	[38]
113	ML-Kem	15.2237	-1.5662	Arid	DBF	NA	-
114	MY-PSO	2.9730	102.3062	Tropical	EBF	106	[42]
115	NL-Loo	52.1666	5.7436	Temperate	ENF	106	[24]
116	PA-SPn	9.3181	-79.6346	Tropical	DBF	7	-
117	PT-Esp	38.6394	-8.6018	Temperate	EBF	12	-
118	PT-Mi1	38.5407	-8.0004	Temperate	EBF	88	[20]
119	RU-Fyo	56.4615	32.9221	Boreal	ENF	236	[48]
120	RU-SkP	62.2550	129.1680	Boreal	DNF	161	-
121	RU-Zot	60.8008	89.3508	Boreal	ENF	201	[79]
122	SD-Dem	13.2829	30.4783	Arid	SAV	NA	-
123	SE-Abi	68.3624	18.7948	Tundra	DBF	NA	[52]
124	SE-Fla	64.1128	19.4569	Boreal	ENF	37	[85]
125	SE-Nor	60.0865	17.4795	Boreal	ENF	105	[49]
126	SE-Sk1	60.1250	17.9181	Boreal	ENF	2	[29]
127	SE-Sk2	60.1297	17.8401	Boreal	ENF	33	[52]
128	SK-Tat	49.1208	20.1635	Boreal	ENF	0	-
129	SN-Dhr	15.4028	-15.4322	Arid	SAV	NA	-
130	UK-Gri	56.6072	-3.7981	Temperate	ENF	21	[55]
131	UK-Ham	51.1208	-0.8608	Temperate	DBF	64	[89]
132	UK-PL3	51.4500	-1.2667	Temperate	DBF	NA	-
133	US-Bar	44.0646	-71.2881	Boreal	DBF	128	[45]
134	US-Blo	38.8953	-120.6328	Temperate	ENF	13	[30]
135	US-Bn1	63.9198	-145.3780	Boreal	ENF	83	[53]
136	US-Bn2	63.9198	-145.3780	Boreal	DBF	16	[53]
137	US-Dk2	35.9736	-79.1004	Temperate	DBF	98	[25]
138	US-Dk3	35.9782	-79.0942	Temperate	ENF	21	[76]
139	US-Fmf	35.1426	-111.7273	Temperate	ENF	150	-
140	US-FR2	29.9495	-97.9962	Temperate	WSA	NA	-

Continued on next page

	Site ID	Lat [°N]	Long [°E]	Climate type	Vegetation type	Forest age	References
141	US-Fuf	35.0890	-111.7620	Temperate	ENF	101	-
142	US-GBT	41.3658	-106.2397	Boreal	ENF	176	-
143	US-GLE	41.3665	-106.2399	Boreal	ENF	184	-
144	US-Ha1	42.5378	-72.1715	Boreal	DBF	96	[83]
145	US-Ha2	42.5393	-72.1779	Boreal	ENF	91	[83]
146	US-Ho1	45.2041	-68.7402	Boreal	ENF	206	[39]
147	US-Ho2	45.2091	-68.7470	Boreal	ENF	208	[39]
148	US-KS1	28.4583	-80.6709	Temperate	ENF	7	[13]
149	US-LPH	42.5419	-72.1850	Boreal	DBF	98	[12]
150	US-Me1	44.5794	-121.5000	Temperate	ENF	2	[50]
151	US-Me2	44.4523	-121.5574	Temperate	ENF	94	[50]
152	US-Me3	44.3154	-121.6078	Temperate	ENF	20	[87]
153	US-Me4	44.4992	-121.6224	Temperate	ENF	24	[50]
154	US-Me5	44.4372	-121.5668	Temperate	ENF	22	[50]
155	US-Me6	44.3233	-121.6078	Temperate	ENF	22	[73]
156	US-MMS	39.3232	-86.4131	Temperate	DBF	95	[71]
157	US-MOz	38.7441	-92.2000	Temperate	DBF	78	[36]
158	US-NC2	35.8031	-76.6679	Temperate	ENF	14	[64]
159	US-NR1	40.0329	-105.5464	Boreal	ENF	110	[58]
160	US-Oho	41.5545	-83.8438	Boreal	DBF	50	[65]
161	US-PFa	45.9459	-90.2723	Boreal	MF	150	[22]
162	US-Prr	65.1237	-147.4876	Boreal	ENF	98	[61]
163	US-SP1	29.7381	-82.2188	Temperate	ENF	63	[16]
164	US-SP2	29.7648	-82.2448	Temperate	ENF	4	[16]
165	US-SP3	29.7548	-82.1633	Temperate	ENF	12	[16]
166	US-SP4	29.8028	-82.2031	Temperate	ENF	0	[16]
167	US-SRM	31.8214	-110.8661	Arid	WSA	201	-
168	US-Syv	46.2420	-89.3477	Boreal	MF	>300	[23]
169	US-Ton	38.4316	-120.9660	Temperate	WSA	NA	-

Continued on next page

	Site ID	Lat [°N]	Long [°E]	Climate type	Vegetation type	Forest age	References
170	US-UMB	45.5598	-84.7138	Boreal	DBF	93	[31]
171	US-UMd	45.5625	-84.6975	Boreal	DBF	90	-
172	US-WBW	35.9588	-84.2874	Temperate	DBF	110	[86]
173	US-WCr	45.8059	-90.0799	Boreal	DBF	96	[23]
174	US-Wi0	46.6188	-91.0814	Boreal	ENF	7	[63]
175	US-Wi1	46.7305	-91.2329	Boreal	DBF	15	[63]
176	US-Wi3	46.6347	-91.0987	Boreal	DBF	66	[63]
177	US-Wi4	46.7393	-91.1663	Boreal	ENF	66	[63]
178	US-Wi5	46.6531	-91.0858	Boreal	ENF	9	[63]
179	US-Wi8	46.7223	-91.2524	Boreal	DBF	2	[63]
180	US-Wi9	46.6188	-91.0814	Boreal	ENF	16	[63]
181	US-Wrc	45.8205	-121.9520	Temperate	ENF	>300	[17]
182	VU-Coc	-15.4427	167.1920	Tropical	EBF	20	[72]
183	ZA-Kru	-25.0197	31.4969	Temperate	SAV	NA	-
184	ZA-Map	-23.8325	31.2144	Temperate	SAV	NA	-
185	ZM-Mon	-15.4378	23.2528	Tropical	WSA	88	-

References

[1] Anthoni, P., Knohl, A., Rebmann, C., Freibauer, A., Mund, M., Ziegler, W., Kolle, O., Schulze, E.-D., 2004. Forest and agricultural land-use-dependent CO_2 exchange in thuringia, germany. Global Change Biology 10 (12), 2005–2019.

[2] Arain, M. A., Restrepo-Coupe, N., 2005. Net ecosystem production in a temperate pine plantation in southeastern canada. Agricultural and Forest Meteorology 128 (3), 223–241.

[3] Araújo, A., Nobre, A., Kruijt, B., Elbers, J., Dallarosa, R., Stefani, P., Von Randow, C., Manzi, A., Culf, A., Gash, J., et al., 2002. Comparative measurements of carbon dioxide fluxes from two nearby towers in a central amazonian rainforest: The manaus lba site. Journal of Geophysical Research: Atmospheres 107 (D20), LBA–58.

[4] Aubinet, M., Chermanne, B., Vandenhaute, M., Longdoz, B., Yernaux, M., Laitat, E., 2001. Long term carbon dioxide exchange above a mixed forest in the belgian ardennes. Agricultural and Forest Meteorology 108 (4), 293–315.

[5] Baldocchi, D. D., Vogel, C. A., Hall, B., 1997. Seasonal variation of carbon dioxide exchange rates above and below a boreal jack pine forest. Agricultural and Forest Meteorology 83 (1), 147–170.

[6] Berbigier, P., Bonnefond, J.-M., Mellmann, P., 2001. CO_2 and water vapour fluxes for 2 years above Euroflux forest site. Agricultural and Forest Meteorology 108 (3), 183–197.

[7] Bergeron, O., Margolis, H. A., Black, T. A., Coursolle, C., Dunn, A. L., Barr, A. G., Wofsy, S. C., 2007. Comparison of carbon dioxide fluxes over three boreal black spruce forests in Canada. Global Change Biology 13 (1), 89–107.

[8] Bernhofer, C., Aubinet, M., Clement, R., Grelle, A., Grünwald, T., Ibrom, A., Jarvis, P., Rebmann, C., Schulze, E.-D., Tenhunen, J., 2003. Spruce forests (norway and sitka spruce, including douglas fir): Carbon and water fluxes and balances, ecological and ecophysiological determinants. Fluxes of carbon, water and energy of European forests. Springer, 99–123.

[9] Bernhofer, C., Gay, L., Granier, A., Joss, U., Kessler, A., Köstner, B., Siegwolf, R., Tenhunen, J. D., Vogt, R., 1996. The hartx-synthesis: an experimental approach to water and carbon exchange of a scots pine plantation. Theoretical and applied climatology 53 (1-3), 173–183.

[10] Black, T., Hartog, G. d., Neumann, H., Blanken, P., Yang, P., Russell, C., Nesic, Z., Lee, X., Chen, S., Staebler, R., et al., 1996. Annual cycles of water vapour and carbon dioxide fluxes in and above a boreal aspen forest. Global Change Biology 2 (3), 219–229.

[11] Bonal, D., Bosc, A., Ponton, S., GORET, J.-Y., Burban, B., Gross, P., BONNEFOND, J.-M., Elbers, J., Longdoz, B., Epron, D., et al., 2008. Impact of severe dry season on net ecosystem exchange in the neotropical rainforest of french guiana. Global Change Biology 14 (8), 1917–1933.

[12] Borken, W., Savage, K., Davidson, E. A., Trumbore, S. E., 2006. Effects of experimental

drought on soil respiration and radiocarbon efflux from a temperate forest soil. Global Change Biology 12 (2), 177–193.

[13] Bracho, R., Powell, T. L., Dore, S., Li, J., Hinkle, C. R., Drake, B. G., 2008. Environmental and biological controls on water and energy exchange in florida scrub oak and pine flatwoods ecosystems. Journal of Geophysical Research: Biogeosciences 113 (G2).

[14] Carswell, F., Costa, A., Palheta, M., Malhi, Y., Meir, P., Costa, J., Ruivo, M. d. L., Leal, L., Costa, J., Clement, R., et al., 2002. Seasonality in CO_2 and H_2O flux at an eastern amazonian rain forest. Journal of Geophysical Research: Atmospheres 107 (D20).

[15] Chiesi, M., Maselli, F., Bindi, M., Fibbi, L., Cherubini, P., Arlotta, E., Tirone, G., Matteucci, G., Seufert, G., 2005. Modelling carbon budget of mediterranean forests using ground and remote sensing measurements. Agricultural and Forest Meteorology 135 (1), 22–34.

[16] Clark, K. L., Gholz, H. L., Moncrieff, J. B., Cropley, F., Loescher, H. W., 1999. Environmental controls over net exchanges of carbon dioxide from contrasting florida ecosystems. Ecological Applications 9 (3), 936–948.

[17] Cook, B. D., Davis, K. J., Wang, W., Desai, A., Berger, B. W., Teclaw, R. M., Martin, J. G., Bolstad, P. V., Bakwin, P. S., Yi, C., et al., 2004. Carbon exchange and venting anomalies in an upland deciduous forest in northern wisconsin, usa. Agricultural and Forest Meteorology 126 (3), 271–295.

[18] Coursolle, C., Margolis, H. A., Barr, A. G., Black, T. A., Amiro, B. D., McCaughey, J. H., Flanagan, L. B., Lafleur, P. M., Roulet, N. T., Bourque, C. P.-A., et al., 2006. Late-summer carbon fluxes from Canadian forests and peatlands along an east west continental transect. Canadian Journal of Forest Research 36 (3), 783–800.

[19] Da Rocha, H. R., Manzi, A. O., Cabral, O. M., Miller, S. D., Goulden, M. L., Saleska, S. R., R-Coupe, N., Wofsy, S. C., Borma, L. S., Artaxo, P., et al., 2009. Patterns of water and heat flux across a biome gradient from tropical forest to savanna in Brazil. Journal of Geophysical Research: Biogeosciences 114 (G1).

[20] David, T., Ferreira, M., Cohen, S., Pereira, J., David, J., 2004. Constraints on tran-

spiration from an evergreen oak tree in southern Portugal. Agricultural and Forest Meteorology 122 (3-4), 193–205.

[21] de Pury, D. G., Ceulemans, R., 1997. Scaling-up carbon fluxes from leaves to stands in a patchy coniferous/deciduous forest. Impacts of Global Change on Tree Physiology and Forest Ecosystems. Springer, 263–272.

[22] Desai, A. R., 2014. Influence and predictive capacity of climate anomalies on daily to decadal extremes in canopy photosynthesis. Photosynthesis research 119 (1-2), 31–47.

[23] Desai, A. R., Bolstad, P. V., Cook, B. D., Davis, K. J., Carey, E. V., 2005. Comparing net ecosystem exchange of carbon dioxide between an old-growth and mature forest in the upper midwest, USA. Agricultural and Forest Meteorology 128 (1), 33–55.

[24] Dolman, A., Moors, E., Elbers, J., 2002. The carbon uptake of a mid latitude pine forest growing on sandy soil. Agricultural and Forest Meteorology 111 (3), 157–170.

[25] Ellsworth, D. S., Reich, P. B., Naumburg, E. S., Koch, G. W., Kubiske, M. E., Smith, S. D., 2004. Photosynthesis, carboxylation and leaf nitrogen responses of 16 species to elevated pCO_2 across four free-air CO_2 enrichment experiments in forest, grassland and desert. Global Change Biology 10 (12), 2121–2138.

[26] Etzold, S., Ruehr, N. K., Zweifel, R., Dobbertin, M., Zingg, A., Pluess, P., Häsler, R., Eugster, W., Buchmann, N., 2011. The carbon balance of two contrasting mountain forest ecosystems in Switzerland: similar annual trends, but seasonal differences. Ecosystems 14 (8), 1289–1309.

[27] Finnigan, J., Leuning, R., 2000. Long term flux measurements-coordinate systems and averaging. In: Proc. International Workshop for Advanced Flux Network and Flux Evaluation. Centre for Global Environmental Research, National Institute for Environmental Studies, 51–56.

[28] Giasson, M.-A., Coursolle, C., Margolis, H. A., 2006. Ecosystem-level CO_2 fluxes from a boreal cutover in eastern Canada before and after scarification. Agricultural and Forest Meteorology 140 (1), 23–40.

[29] Gioli, B., Miglietta, F., De Martino, B., Hutjes, R. W., Dolman, H. A., Lindroth, A., Schumacher, M., Sanz, M. J., Manca, G., Peressotti, A., et al., 2004. Comparison between tower and aircraft-based eddy covariance fluxes in five European regions. Agricultural and Forest Meteorology 127 (1), 1–16.

[30] Goldstein, A., Hultman, N., Fracheboud, J., Bauer, M., Panek, J., Xu, M., Qi, Y., Guenther, A., Baugh, W., 2000. Effects of climate variability on the carbon dioxide, water, and sensible heat fluxes above a ponderosa pine plantation in the Sierra Nevada (ca). Agricultural and Forest Meteorology 101 (2), 113–129.

[31] Gough, C., Vogel, C., Schmid, H., Su, H.-B., Curtis, P., 2008. Multi-year convergence of biometric and meteorological estimates of forest carbon storage. Agricultural and Forest Meteorology 148 (2), 158–170.

[32] Goulden, M. L., Winston, G. C., McMILLAN, A., Litvak, M. E., Read, E. L., Rocha, A. V., Rob Elliot, J., 2006. An eddy covariance mesonet to measure the effect of forest age on land–atmosphere exchange. Global Change Biology 12 (11), 2146–2162.

[33] Gower, S., Vogel, J., Stow, T., Norman, J., Steele, S., Kucharik, C., 1997. Carbon distribution and above-ground net primary production of upland and lowland boreal forests in Saskatchewan and Manitoba. Journal of Geophysical Research 102 (D24), 29029–41.

[34] Granier, A., Ceschia, E., Damesin, C., Dufrêne, E., Epron, D., Gross, P., Lebaube, S., Le Dantec, V., Le Goff, N., Lemoine, D., et al., 2000. The carbon balance of a young beech forest. Functional ecology 14 (3), 312–325.

[35] Grünzweig, J., Lin, T., Rotenberg, E., Schwartz, A., Yakir, D., 2003. Carbon sequestration in arid-land forest. Global Change Biology 9 (5), 791–799.

[36] Gu, L., Meyers, T., Pallardy, S. G., Hanson, P. J., Yang, B., Heuer, M., Hosman, K. P., Liu, Q., Riggs, J. S., Sluss, D., et al., 2007. Influences of biomass heat and biochemical energy storages on the land surface fluxes and radiative temperature. Journal of Geophysical Research: Atmospheres 112 (D2).

[37] Guan, D.-X., Wu, J.-B., Zhao, X.-S., Han, S.-J., Yu, G.-R., Sun, X.-M., Jin, C.-J., 2006.

Co 2 fluxes over an old, temperate mixed forest in northeastern China. Agricultural and Forest Meteorology 137 (3), 138–149.

[38] Hirano, T., Hirata, R., Fujinuma, Y., Saigusa, N., Yamamoto, S., Harazono, Y., Takada, M., Inukai, K., Inoue, G., 2003. Co2 and water vapor exchange of a larch forest in northern Japan. Tellus B 55 (2), 244–257.

[39] Hollinger, D., Aber, J., Dail, B., Davidson, E., Goltz, S., Hughes, H., Leclerc, M., Lee, J., Richardson, A., Rodrigues, C., et al., 2004. Spatial and temporal variability in forest–atmosphere CO_2 exchange. Global Change Biology 10 (10), 1689–1706.

[40] Howard, E. A., Gower, S. T., Foley, J. A., Kucharik, C. J., 2004. Effects of logging on carbon dynamics of a jack pine forest in Saskatchewan, Canada. Global Change Biology 10 (8), 1267–1284.

[41] Humphreys, E. R., Black, T. A., Morgenstern, K., Cai, T., Drewitt, G. B., Nesic, Z., Trofymow, J., 2006. Carbon dioxide fluxes in coastal douglas-fir stands at different stages of development after clearcut harvesting. Agricultural and Forest Meteorology 140 (1), 6–22.

[42] Ito, A., Oikawa, T., 2002. A simulation model of the carbon cycle in land ecosystems (sim-cycle): a description based on dry-matter production theory and plot-scale validation. Ecological Modelling 151 (2), 143–176.

[43] Jarvis, P., Massheder, J., Hale, S., Moncrieff, J., Rayment, M., Scott, S., 1997. Seasonal variation of carbon dioxide, water vapor, and energy exchanges of a boreal black spruce forest. Journal of Geophysical Research: Atmospheres 102 (D24), 28953–28966.

[44] Jassal, R. S., Black, T. A., Novak, M. D., GAUMONT-GUAY, D., Nesic, Z., 2008. Effect of soil water stress on soil respiration and its temperature sensitivity in an 18-year-old temperate douglas-fir stand. Global Change Biology 14 (6), 1305–1318.

[45] Jenkins, J., Richardson, A., Braswell, B., Ollinger, S., Hollinger, D., Smith, M.-L., 2007. Refining light-use efficiency calculations for a deciduous forest canopy using simultaneous tower-based carbon flux and radiometric measurements. Agricultural and Forest Meteorology 143 (1), 64–79.

[46] Joiner, D. W., McCaughey, J. H., Lafleur, P. M., Bartlett, P. A., 1999. Water and carbon dioxide exchange at a boreal young jack pine forest in the boreas northern study area. Journal of Geophysical Research: Atmospheres 104 (D22), 27641–27652.

[47] Knohl, A., Schulze, E.-D., Kolle, O., Buchmann, N., 2003. Large carbon uptake by an unmanaged 250-year-old deciduous forest in central Germany. Agricultural and Forest Meteorology 118 (3), 151–167.

[48] Kurbatova, J., Li, C., Varlagin, A., Xiao, X., Vygodskaya, N., 2008. Modeling carbon dynamics in two adjacent spruce forests with different soil conditions in Russia. Biogeosciences 5 (4), 969–980.

[49] Lagergren, F., Lindroth, A., Dellwik, E., Ibrom, A., Lankreijer, H., Launiainen, S., Mölder, M., Kolari, P., Pilegaard, K., Vesala, T., 2008. Biophysical controls on CO_2 fluxes of three northern forests based on long-term eddy covariance data. Tellus B 60 (2), 143–152.

[50] Law, B. E., Sun, O., Campbell, J., Van Tuyl, S., Thornton, P., 2003. Changes in carbon storage and fluxes in a chronosequence of ponderosa pine. Global Change Biology 9 (4), 510–524.

[51] Lindauer, M., Schmid, H., Grote, R., Mauder, M., Steinbrecher, R., Wolpert, B., 2014. Net ecosystem exchange over a non-cleared wind-throw-disturbed upland spruce forest-measurements and simulations. Agricultural and Forest Meteorology 197, 219–234.

[52] Lindroth, A., Lagergren, F., Aurela, M., Bjarnadottir, B., Christensen, T., Dellwik, E., Grelle, A., Ibrom, A., Johansson, T., Lankreijer, H., et al., 2008. Leaf area index is the principal scaling parameter for both gross photosynthesis and ecosystem respiration of northern deciduous and coniferous forests. Tellus B: Chemical and Physical Meteorology 60 (2), 129–142.

[53] Liu, H., Randerson, J. T., Lindfors, J., Chapin, F. S., 2005. Changes in the surface energy budget after fire in boreal ecosystems of interior Alaska: An annual perspective. Journal of Geophysical Research: Atmospheres 110 (D13).

[54] Maselli, F., Barbati, A., Chiesi, M., Chirici, G., Corona, P., 2006. Use of remotely

sensed and ancillary data for estimating forest gross primary productivity in Italy. Remote Sensing of Environment 100 (4), 563–575.

[55] Medlyn, B. E., Robinson, A. P., Clement, R., McMurtrie, R. E., 2005. On the validation of models of forest co2 exchange using eddy covariance data: some perils and pitfalls. Tree Physiology 25 (7), 839–857.

[56] Michelot, A., Eglin, T., Dufrene, E., Lelarge-Trouverie, C., Damesin, C., 2011. Comparison of seasonal variations in water-use efficiency calculated from the carbon isotope composition of tree rings and flux data in a temperate forest. Plant, cell & environment 34 (2), 230–244.

[57] Migliavacca, M., Meroni, M., Manca, G., Matteucci, G., Montagnani, L., Grassi, G., Zenone, T., Teobaldelli, M., Goded, I., Colombo, R., et al., 2009. Seasonal and interannual patterns of carbon and water fluxes of a poplar plantation under peculiar eco-climatic conditions. Agricultural and Forest Meteorology 149 (9), 1460–1476.

[58] Monson, R., Turnipseed, A., Sparks, J., Harley, P., Scott-Denton, L., Sparks, K., Huxman, T., 2002. Carbon sequestration in a high-elevation, subalpine forest. Global Change Biology 8 (5), 459–478.

[59] Montagnani, L., Manca, G., Canepa, E., Georgieva, E., Acosta, M., Feigenwinter, C., Janous, D., Kerschbaumer, G., Lindroth, A., Minach, L., et al., 2009. A new mass conservation approach to the study of CO_2 advection in an alpine forest. Journal of Geophysical Research: Atmospheres 114 (D7).

[60] Morgenstern, K., Black, T. A., Humphreys, E. R., Griffis, T. J., Drewitt, G. B., Cai, T., Nesic, Z., Spittlehouse, D. L., Livingston, N. J., 2004. Sensitivity and uncertainty of the carbon balance of a pacific northwest douglas-fir forest during an el niño/la niña cycle. Agricultural and Forest Meteorology 123 (3), 201–219.

[61] Nakai, T., Kim, Y., Busey, R. C., Suzuki, R., Nagai, S., Kobayashi, H., Park, H., Sugiura, K., Ito, A., 2013. Characteristics of evapotranspiration from a permafrost black spruce forest in interior Alaska. Polar Science 7 (2), 136–148.

[62] Nardino, M., Georgiadis, T., Rossi, F., Ponti, F., Miglietta, F., Magliulo, V., 2002.

Primary productivity and evapotranspiration of a mixed forest. Congress CNR-ISA Fo., Istituto per i Sistemi Agricoli e Forestali del Mediterraneo, Portici. 24–25.

[63] Noormets, A., Chen, J., Crow, T. R., 2007. Age-dependent changes in ecosystem carbon fluxes in managed forests in northern Wisconsin, USA. Ecosystems 10 (2), 187–203.

[64] Noormets, A., Gavazzi, M. J., McNulty, S. G., DOMEC, J.-C., Sun, G., King, J. S., Chen, J., 2010. Response of carbon fluxes to drought in a coastal plain loblolly pine forest. Global Change Biology 16 (1), 272–287.

[65] Noormets, A., McNulty, S. G., DeForest, J. L., Sun, G., Li, Q., Chen, J., 2008. Drought during canopy development has lasting effect on annual carbon balance in a deciduous temperate forest. New Phytologist 179 (3), 818–828.

[66] Pejam, M., Arain, M., McCaughey, J., 2006. Energy and water vapour exchanges over a mixedwood boreal forest in ontario, Canada. Hydrological Processes 20 (17), 3709–3724.

[67] Pilegaard, K., Hummelshøj, P., Jensen, N., Chen, Z., 2001. Two years of continuous CO_2 eddy-flux measurements over a Danish beech forest. Agricultural and Forest Meteorology 107 (1), 29–41.

[68] Rambal, S., Joffre, R., Ourcival, J., Cavender-Bares, J., Rocheteau, A., 2004. The growth respiration component in eddy CO_2 flux from a quercus ilex mediterranean forest. Global Change Biology 10 (9), 1460–1469.

[69] Reichstein, M., Falge, E., Baldocchi, D., Papale, D., Aubinet, M., Berbigier, P., Bernhofer, C., Buchmann, N., Gilmanov, T., Granier, A., et al., 2005. On the separation of net ecosystem exchange into assimilation and ecosystem respiration: review and improved algorithm. Global Change Biology 11 (9), 1424–1439.

[70] Rey, A., Pegoraro, E., Tedeschi, V., De Parri, I., Jarvis, P. G., Valentini, R., 2002. Annual variation in soil respiration and its components in a coppice oak forest in central Italy. Global Change Biology 8 (9), 851–866.

[71] Roman, D., Novick, K., Brzostek, E., Dragoni, D., Rahman, F., Phillips, R., 2015. The role of isohydric and anisohydric species in determining ecosystem-scale response to severe drought. Oecologia 179 (3), 641–654.

[72] Roupsard, O., Bonnefond, J.-M., Irvine, M., Berbigier, P., Nouvellon, Y., Dauzat, J., Taga, S., Hamel, O., Jourdan, C., Saint-André, L., et al., 2006. Partitioning energy and evapo-transpiration above and below a tropical palm canopy. Agricultural and Forest Meteorology 139 (3), 252–268.

[73] Ruehr, N. K., Martin, J. G., Law, B. E., 2012. Effects of water availability on carbon and water exchange in a young ponderosa pine forest: Above-and belowground responses. Agricultural and forest meteorology 164, 136–148.

[74] Saleska, S. R., Miller, S. D., Matross, D. M., Goulden, M. L., Wofsy, S. C., Da Rocha, H. R., De Camargo, P. B., Crill, P., Daube, B. C., De Freitas, H. C., et al., 2003. Carbon in amazon forests: unexpected seasonal fluxes and disturbance-induced losses. Science 302 (5650), 1554–1557.

[75] Sanz, M., Carrara, A., Gimeno, C., Bucher, A., Lopez, R., 2004. Effects of a dry and warm summer conditions on CO_2 and energy fluxes from three mediterranean ecosystems. Geophysical Research Abstracts, 6.3239–3239.

[76] Stoy, P. C., Katul, G. G., Siqueira, M., JUANG, J.-Y., Novick, K. A., McCARTHY, H. R., Oishi, A. C., Oren, R., 2008. Role of vegetation in determining carbon sequestration along ecological succession in the southeastern united states. Global Change Biology 14 (6), 1409–1427.

[77] Suni, T., Rinne, J., Reissell, A., Altimir, N., Keronen, P., Rannik, U., Maso, M., Kulmala, M., Vesala, T., 2003. Long-term measurements of surface fluxes above a scots pine forest in hyytiala, southern Finland, 1996-2001. Boreal Environment Research 8 (4), 287–302.

[78] Takagi, K., Fukuzawa, K., Liang, N., Kayama, M., Nomura, M., Hojyo, H., Sugata, S., Shibata, H., Fukazawa, T., Takahashi, Y., et al., 2009. Change in CO_2 balance under a series of forestry activities in a cool-temperate mixed forest with dense undergrowth. Global Change Biology 15 (5), 1275–1288.

[79] Tanja, S., Berninger, F., Vesala, T., Markkanen, T., Hari, P., Mäkelä, A., Ilvesniemi, H., Hänninen, H., Nikinmaa, E., Huttula, T., et al., 2003. Air temperature triggers the recovery of evergreen boreal forest photosynthesis in spring. Global change biology 9 (10), 1410–1426.

[80] Tedeschi, V., Rey, A., Manca, G., Valentini, R., Jarvis, P. G., Borghetti, M., 2006. Soil respiration in a mediterranean oak forest at different developmental stages after coppicing. Global Change Biology 12 (1), 110–121.

[81] Thum, T., Aalto, T., Laurila, T., Aurela, M., Kolari, P., Hari, P., 2007. Parametrization of two photosynthesis models at the canopy scale in a northern boreal scots pine forest. Tellus B 59 (5), 874–890.

[82] Tirone, G., Dore, S., Matteucci, G., Greco, S., Valentini, R., 2003. Evergreen mediterranean forests. carbon and water fluxes, balances, ecological and ecophysiological determinants. Fluxes of Carbon, Water and Energy of European Forests. Springer, 125–149.

[83] Urbanski, S., Barford, C., Wofsy, S., Kucharik, C., Pyle, E., Budney, J., McKain, K., Fitzjarrald, D., Czikowsky, M., Munger, J., 2007. Factors controlling CO_2 exchange on timescales from hourly to decadal at harvard forest. Journal of Geophysical Research: Biogeosciences 112 (G2).

[84] Valentini, R., Angelis, P. d., Matteucci, G., Monaco, R., Dore, S., Mucnozza, G. S., 1996. Seasonal net carbon dioxide exchange of a beech forest with the atmosphere. Global Change Biology 2 (3), 199–207.

[85] Valentini, R., Matteucci, G., Dolman, A., Schulze, E.-D., Rebmann, C., Moors, E., Granier, A., Gross, P., Jensen, N., Pilegaard, K., et al., 2000. Respiration as the main determinant of carbon balance in european forests. Nature 404 (6780), 861–865.

[86] Verma, S. B., Baldocchi, D. D., Anderson, D. E., Matt, D. R., Clement, R. J., 1986. Eddy fluxes of CO_2, water vapor, and sensible heat over a deciduous forest. Boundary-Layer Meteorology 36 (1-2), 71–91.

[87] Vickers, D., Thomas, C., Law, B. E., 2009. Random and systematic CO_2 flux sampling errors for tower measurements over forests in the convective boundary layer. Agricultural and Forest Meteorology 149 (1), 73–83.

[88] von Randow, C., Manzi, A. O., Kruijt, B., De Oliveira, P., Zanchi, F., Silva, R., Hodnett, M., Gash, J., Elbers, J., Waterloo, M., et al., 2004. Comparative measurements and seasonal variations in energy and carbon exchange over forest and pasture in south west amazonia. Theoretical and Applied Climatology 78 (1-3), 5–26.

[89] Wilkinson, M., Eaton, E., Broadmeadow, M., Morison, J., 2012. Inter-annual variation of carbon uptake by a plantation oak woodland in south-eastern England. Biogeosciences 9 (12), 5373–5389.

[90] Yamamoto, S., Murayama, S., Saigusa, N., Kondo, H., 1999. Seasonal and inter-annual variation of co2 flux between a temperate forest and the atmosphere in japan. Tellus B 51 (2), 402–413.

[91] Zhang, L., Luo, Y., Yu, G., Zhang, L., 2010. Estimated carbon residence times in three forest ecosystems of eastern china: Applications of probabilistic inversion. Journal of Geophysical Research: Biogeosciences 115 (G1).

[92] Zielis, S., Etzold, S., Zweifel, R., Eugster, W., Haeni, M., Buchmann, N., 2014. NEP of a Swiss subalpine forest is significantly driven not only by current but also by previous year's weather. Biogeosciences 11 (6), 1627.
