# Supplementary material for: Memory effects of climate and vegetation affecting net ecosystem CO2 fluxes in global forests
Source: PLoS One. 2019 Feb 6;14(2):e0211510. doi: 10.1371/journal.pone.0211510 (PMC6364965; doi:10.1371/journal.pone.0211510)
Supplement: S2 Table — Statistics for the anomalies were not calculated in the arid and tropical climate (i.e. NA) because there was no site with at least 2 years of complete data after data quality control. (PDF) [file pone.0211510.s002.pdf]

S2 Table. RMSE of the *LSTM* setup per PFT and climate region from the ensemble mean mean ±sd estimate of the 50 runs. Statistics for the anomalies were not calculated in the arid and tropical climate (i.e. NA) because there was no site with at least 2 years of complete data after data quality control.

	Seasonal cycle	Seasonal anomaly	Across-site	Interannual anomaly
Deciduous forest	**1.18** ±0.03	**0.61** ±0.01	**0.51** ±0.02	**0.27** ±0.007
Evergreen forest	**1.15** ±0.02	**0.58** ±0.006	**0.72** ±0.03	**0.31** ±0.006
Mixed forest	**1.10** ±0.05	**0.68** ±0.007	**0.50** ±0.08	**0.37** ±0.008
Savanna	**0.75** ±0.02	**0.58** ±0.007	**0.34** ±0.09	**0.19** ±0.01
Arid	**0.71** ±0.03	NA	**0.20** ±0.07	NA
Boreal	**1.02** ±0.02	**0.45** ±0.006	**0.52** ±0.03	**0.18** ±0.004
Temperate	**1.18** ±0.02	**0.71** ±0.006	**0.65** ±0.03	**0.38** ±0.006
Tropical	**1.40** ±0.08	NA	**1.03** ±0.12	NA
