# Supplementary material for: Memory effects of climate and vegetation affecting net ecosystem CO2 fluxes in global forests
Source: PLoS One. 2019 Feb 6;14(2):e0211510. doi: 10.1371/journal.pone.0211510 (PMC6364965; doi:10.1371/journal.pone.0211510)
Supplement: S3 Table — Statistics for the anomalies were not calculated in the arid and tropical climate (i.e. NA) because there was no site with at least 2 years of complete data after data quality control. (PDF) [file pone.0211510.s003.pdf]

S3 Table. MAE of the *LSTM* setup per PFT and climate region from the ensemble mean mean ±sd estimate of the 50 runs. Statistics for the anomalies were not calculated in the arid and tropical climate (i.e. NA) because there was no site with at least 2 years of complete data after data quality control.

	Seasonal cycle	Seasonal anomaly	Across-site	Interannual anomaly
Deciduous forest	**0.86** ± 0.03	**0.42** ± 0.007	**0.45** ± 0.02	**0.20** ± 0.006
Evergreen forest	**0.83** ± 0.02	**0.40** ± 0.004	**0.54** ± 0.03	**0.20** ± 0.004
Mixed forest	**0.78** ± 0.05	**0.47** ± 0.006	**0.43** ± 0.05	**0.29** ± 0.006
Savanna	**0.57** ±0.02	**0.46** ±0.007	**0.22** ±0.06	**0.16** ±0.01
Arid	**0.50** ± 0.03	NA	**0.16** ±0.06	NA
Boreal	**0.68** ± 0.02	**0.30** ± 0.004	**0.37** ±0.02	**0.14** ±0.003
Temperate	**0.89** ± 0.02	**0.51** ± 0.005	**0.54** ±0.02	**0.28** ±0.005
Tropical	**1.12** ± 0.07	NA	**0.85** ±0.10	NA
