# Supplementary material for: Memory effects of climate and vegetation affecting net ecosystem CO2 fluxes in global forests
Source: PLoS One. 2019 Feb 6;14(2):e0211510. doi: 10.1371/journal.pone.0211510 (PMC6364965; doi:10.1371/journal.pone.0211510)
Supplement: S5 Table — LSTM = LSTM model using the full depth of the Landsat time series and climate data; LSTMperm = LSTM model but the temporal patterns of both the predictive and the target variables were randomly permuted while instantaneous relationships between predictive and target variables were kept; LSTMmsc = LSTM model but the Landsat time series for each band were replaced by their mean seasonal cycle, while using the actual values of air temperature (Tair), precipitation (P), global radiation (Rg), and vapor pressure deficit (VPD); LSTMannual = LSTM model but the Landsat time series for each band were replaced by their annual mean, while using the actual values of Tair, P, Rg, and VPD, RF = Random Forest model using the actual values of the Landsat time series and climate data. (PDF) [file pone.0211510.s005.pdf]

S5 Table. RMSE of our proposed approach against the other model set-ups from the ensemble mean mean ±sd estimate of the 50 runs. $LSTM$ = LSTM model using the full depth of the Landsat time series and climate data; $LSTM_{perm}$ = $LSTM$ model but the temporal patterns of both the predictive and the target variables were randomly permuted while instantaneous relationships between predictive and target variables were kept; $LSTM_{msc}$ = $LSTM$ model but the Landsat time series for each band were replaced by their mean seasonal cycle, while using the actual values of air temperature (T_{air}), precipitation (P), global radiation (Rg), and vapor pressure deficit (VPD); $LSTM_{annual}$ = $LSTM$ model but the Landsat time series for each band were replaced by their annual mean, while using the actual values of T_{air}, P, Rg, and VPD, RF = Random Forest model using the actual values of the Landsat time series and climate data.

	Seasonal	Seasonal anomaly	Across-site	Interannual anomaly
LSTM	**1.12** ±0.02	**0.61** ±0.005	**0.63** ±0.02	**0.31** ±0.004
$LSTM_{msc}$	**1.15** ±0.01	**0.61** ±0.003	**0.65** ±0.02	**0.32** ±0.002
$LSTM_{annual}$	**1.23** ±0.03	**0.61** ±0.008	**0.66** ±0.02	**0.32** ±0.008
$LSTM_{perm}$	**1.19** ±0.02	**0.63** ±0.005	**0.65** ±0.03	**0.31** ±0.004
RF	**1.25** ±0.00006	**0.72** ±0.0002	**0.65** ±0.0001	**0.33** ±0.0001
