# Supplementary material for: Memory effects of climate and vegetation affecting net ecosystem CO2 fluxes in global forests
Source: PLoS One. 2019 Feb 6;14(2):e0211510. doi: 10.1371/journal.pone.0211510 (PMC6364965; doi:10.1371/journal.pone.0211510)
Supplement: S1 Fig — The model was trained on 70% of the data and evaluated on 30% of the left out data. nir = near-infrared, swir1 = shortwave infrared 1, swir2 = shortwave infrared 2, and tir = thermal infrared. (PDF) [file pone.0211510.s007.pdf]

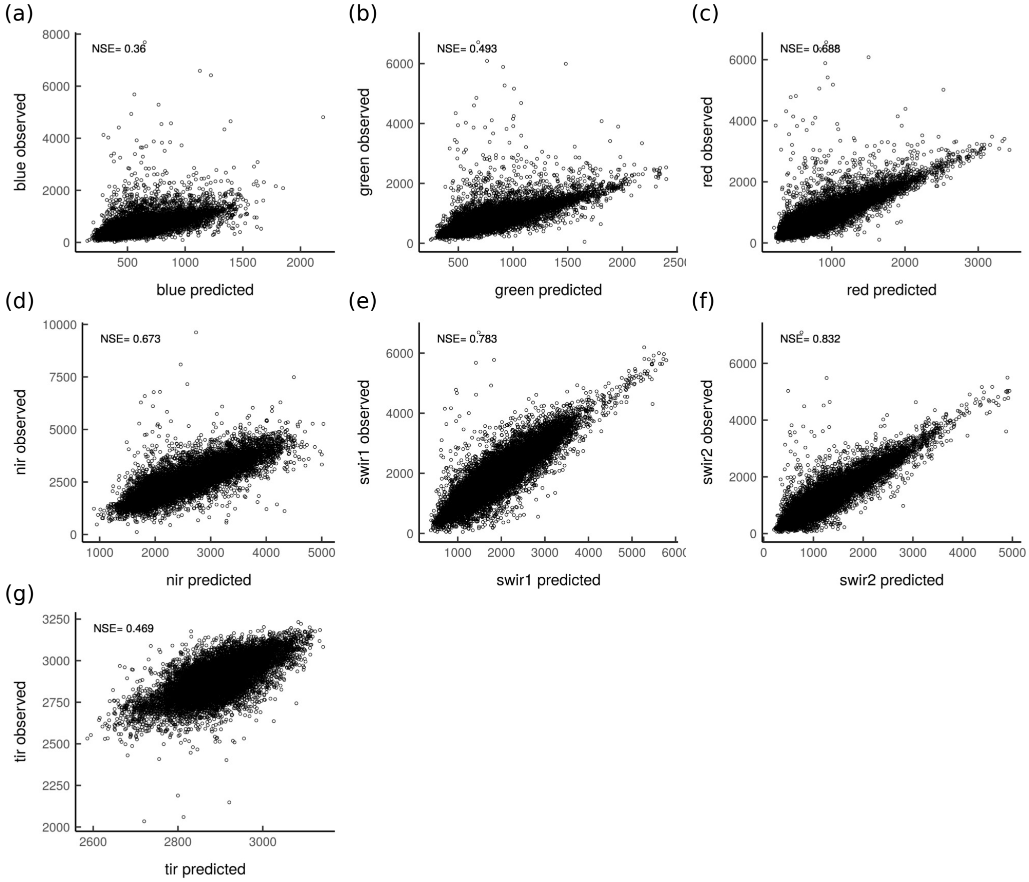

S1 Fig. Performance of the gap-filling procedure of each Landsat band using a Random Forest model and the MODIS bands as predictive variables. The model was trained on 70% of the data and evaluated on 30% of the left out data. nir = near-infrared, swir1 = shortwave infrared 1, swir2 = shortwave infrared 2, and tir = thermal infrared.
