# Supplementary material for: Memory effects of climate and vegetation affecting net ecosystem CO2 fluxes in global forests
Source: PLoS One. 2019 Feb 6;14(2):e0211510. doi: 10.1371/journal.pone.0211510 (PMC6364965; doi:10.1371/journal.pone.0211510)
Supplement: S3 Fig — Assessment of the gap-filling procedure was done for Tair, Precip, Rg, and VPD. For Tair, Rg, and VPD, the Nash-Sutcliffe efficiency (NSE) is reported, while the root mean squared error (RMSE) is reported for Precip. (PDF) [file pone.0211510.s009.pdf]

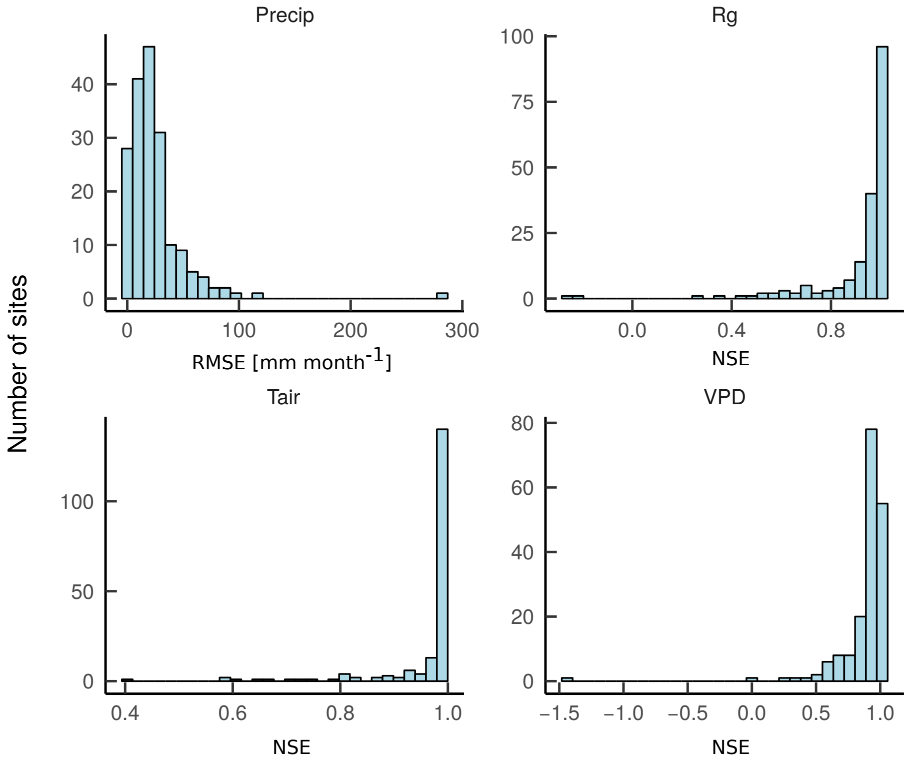

S3 Fig. Performance of the gap-filling procedure for the differtent climate variables. Assessment of the gap-filling procedure was done for T_{air}, Precip, Rg, and VPD. For T_{air}, Rg, and VPD, the Nash-Sutcliffe efficiency (NSE) is reported, while the root mean squared error (RMSE) is reported for Precip.
