# Supplementary material for: Memory effects of climate and vegetation affecting net ecosystem CO2 fluxes in global forests
Source: PLoS One. 2019 Feb 6;14(2):e0211510. doi: 10.1371/journal.pone.0211510 (PMC6364965; doi:10.1371/journal.pone.0211510)
Supplement: S4 Fig — The coefficient of determination was computed using monthly observed and predicted NEE estimates for each site. Each point represents one site and only the sites with at least one complete year of good quality data (n site = 81) are shown. (PDF) [file pone.0211510.s010.pdf]

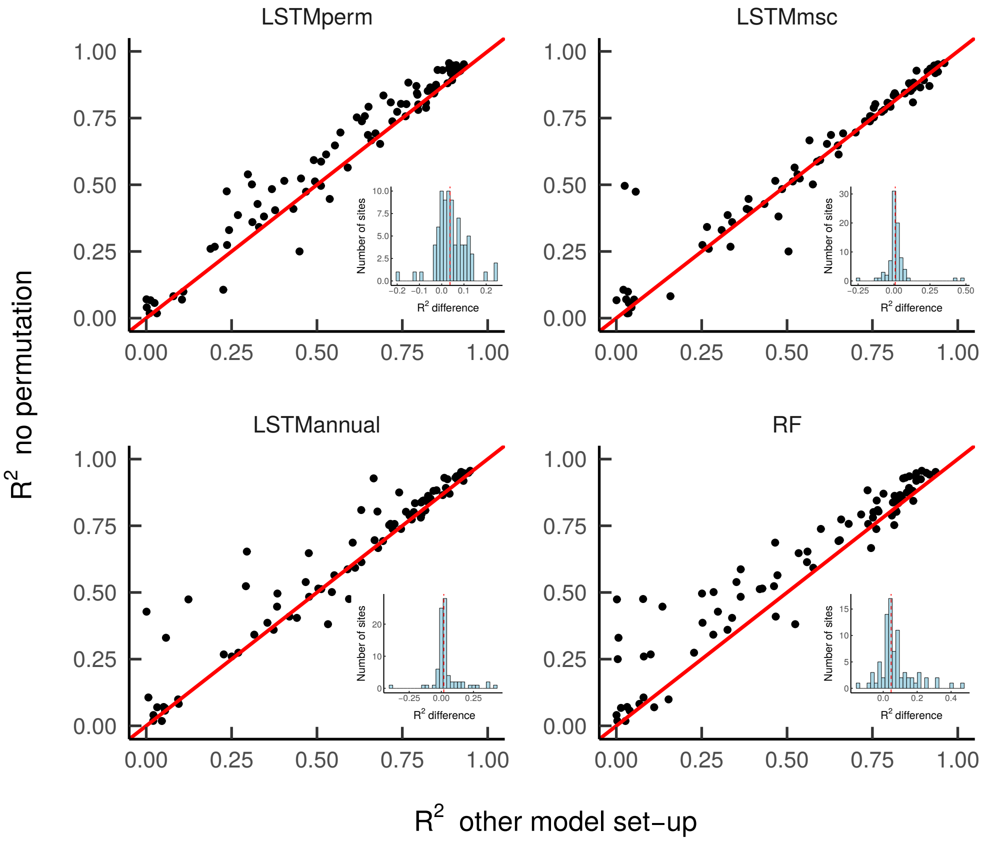

S4 Fig. Scatterplots of the coefficient of determination of the proposed approach against the other model set-ups at site level. The coefficient of determination was computed using monthly observed and predicted NEE estimates for each site. Each point represents one site and only the sites with at least one complete year of good quality data (n site = 81) are shown.
